# Multiyear Assessment of Biofertilizer Application on ‘Gala’ Apple Orchards: Impacts on Soil Fertility, Leaf Mineral Content, and Agronomic Performance

**DOI:** 10.3390/plants14213319

**Published:** 2025-10-30

**Authors:** Susana Ferreira, Catarina Lopes, Marta Gonçalves, Margarida Rodrigues, Francisco Martinho, Miguel Leão de Sousa

**Affiliations:** 1 National Institute for Agrarian and Veterinary Research (INIAV), I.P., Estrada de Leiria, 2460-059 Alcobaça, Portugal; marta.goncalves@iniav.pt (M.G.); margarida.rodrigues@iniav.pt (M.R.); francisco.martinho@iniav.pt (F.M.); 2 Instituto de Desarrollo Regional, Universidad de Castilla-La Mancha (UCLM), 02071 Albacete, Spain; 3 Department of Conservation Biology, Estación Biológica de Doñana (EBD-CSIC), Avenida Americo Vespucio 26, 41092 Sevilla, Spain; catarina.santos@ebd.csic.es; 4 GREEN-IT—Bioresources for Sustainability, 2780-157 Oeiras, Portugal

**Keywords:** fertilizer strategies, leaf analysis, *Malus domestica* Borkh, mineral soil dynamics, sustainability, tree and fruit growth, yield

## Abstract

Biofertilizers are sustainable alternatives to mineral fertilizers in perennial crops, reducing the need for mineral inputs. This five-year field study evaluated three biofertilizers—Mycoshell^®^ (*Glomus* spp. + humic/fulvic acids), Kiplant iNmass^®^ (*Azospirillum brasilense*, *Bacillus megaterium*, *and Saccharomyces cerevisiae*), and Kiplant All-Grip^®^ (*Bacillus megaterium* and *Pseudomonas* spp.)—at different dosages alongside two mineral fertilizer regimes, T100 (full recommended dose) and T70 (70% of T100, alone or combined with biofertilizers), in an apple orchard under Mediterranean conditions. Biofertilizers maintained or increased soil nutrient availability by 5–15% and leaf N, P, K, Mg, and Zn concentrations by 5–12% compared with T100. Trees under biofertilizers, particularly Myc2 and Myc4, exhibited greater shoot growth (up to 30.4 m/year), trunk cross-sectional area (TCSA: 11.9 cm^2^ in 2022), and canopy volume (2.21 m^3^), representing 10–20% increases. Selected biofertilizer treatments produced 6–7.5 kg/tree, 130–145 g average fruit weight, 66–74 mm diameter, 13.9–18.7 °Brix, and 13–18% dry matter, maintaining >90% of yield and fruit size relative to T100, with more balanced medium- and large-sized fruit distribution. Principal Component Analysis explained 66–72% of soil and leaf nutrient variance, confirming their multivariate impact. Overall, biofertilizers applied at recommended doses and timings can partially replace mineral fertilizers, sustaining productivity and quality, enhancing nutrient availability, and supporting long-term orchard sustainability. While climate remains the main driver of annual production, these findings provide evidence for integrating biofertilizers into environmentally friendly fertilization strategies.

## 1. Introduction

The transition toward sustainable agriculture increasingly emphasizes the adoption of environmentally friendly alternatives to conventional agrochemicals, including biofertilizers. Excessive reliance on synthetic fertilizers can degrade soil physical and chemical properties and disrupt nutrient cycling. This may lead to environmental consequences, including eutrophication, greenhouse gas emissions, and heavy metal accumulation, ultimately compromising long-term soil fertility and crop resilience [1,2,3].

Biofertilizers—microbial formulations containing beneficial bacteria and fungi—offer an eco-efficient approach to improving nutrient availability, stimulating plant growth, and enhancing soil health while mitigating these negative impacts [4,5,6,7]. They act through multiple mechanisms, including biological nitrogen fixation, phosphorus solubilization, potassium mobilization, phytohormone modulation, pathogen suppression, and improved tolerance to abiotic stresses [8,9,10]. Biofertilizers are categorized according to the microorganisms they harbor and their specific contributions to plant nutrition: (i) nitrogen-fixing biofertilizers contain microbes capable of converting atmospheric nitrogen into plant-available forms, including *Rhizobium*, *Azotobacter*, *Azospirillum*, and cyanobacteria; (ii) phosphate-solubilizing biofertilizers host microorganisms such as *Bacillus*, *Pseudomonas*, and *Aspergillus* that solubilize insoluble phosphates; (iii) phosphate-mobilizing biofertilizers comprise mycorrhizal fungi that facilitate phosphorus uptake from the soil; (iv) potassium-solubilizing biofertilizers include species like *Bacillus mucilaginosus* and *Bacillus edaphicus* that release potassium from mineral sources; (v) sulfur-oxidizing biofertilizers contain microbes such as *Thiobacillus* that oxidize sulfur to forms plants can use; and (vi) plant growth-promoting rhizobacteria (PGPR) provide a range of benefits through hormone synthesis, siderophore production, and pathogen suppression [11,12,13]. Symbiotic and associative interactions with the host plant can enhance nutrient acquisition, stimulate root development, and improve physiological performance under variable environmental conditions [14,15,16].

The mechanisms of these biofertilizers are expected to interact with orchard-specific sandy-loam soils and the Mediterranean climate, where nutrient availability and water limitations significantly influence plant growth and yield. Field application of biofertilizers may face challenges such as inconsistent microbial establishment, sensitivity to environmental stresses, and variability in nutrient release, which must be considered when evaluating long-term effects. Additionally, while biofertilizer adoption can reduce chemical inputs and provide environmental benefits, potential trade-offs such as initial cost, variability in efficacy, and labor requirements should be critically assessed.

Although biofertilizers have been widely studied in annual crops, there is a scarcity of long-term, multi-year trials in Mediterranean apple orchards, particularly evaluating cumulative effects on soil properties and crop performance under water-limited conditions. Previous multi-year studies on perennial crops have reported inconsistent effects of biofertilizers on yield and soil fertility, with some trials showing strong benefits while others observed limited or variable responses [17,18,19]. These discrepancies highlight the need for locally adapted, long-term field experiments.

The ‘Gala’ apple cultivar was selected due to its economic importance, its inclusion in the Maçã de Alcobaça Protected Geographical Indication (IGP), and its relevance for local orchard management practices under Mediterranean conditions. Multi-year experiments are essential to capture temporal dynamics, treatment stability, and cumulative effects on soil chemical properties, leaf nutrient composition, vegetative growth, fruit set, yield, and fruit quality [20,21,22,23,24,25]. Improvements in soil fertility parameters, such as available nitrogen and phosphorus, are expected to translate into measurable changes in leaf nutrient content, vegetative growth, and fruit yield, providing integrated indicators of biofertilizer effectiveness.

This study addresses the identified knowledge gap by monitoring orchard responses over five consecutive years, providing robust insights into treatment performance and stability. The research is framed within the EU Farm to Fork strategy and broader Mediterranean sustainability initiatives, which emphasize reducing chemical inputs, enhancing nutrient use efficiency, and promoting resilient cropping systems [26,27,28]. Despite these strategic priorities, empirical long-term data on biofertilizer performance in Mediterranean apple orchards remain limited, particularly under water-limited conditions, highlighting the need for locally adapted field trials [20,21,22]. This article represents the first paper of a two-part series based on a comprehensive biofertilizer field experiment in ‘Gala’ apple orchards in western Portugal. The present study focuses on soil fertility, leaf mineral content, and agronomic performance, while a companion paper will explore physiological and mechanistic responses under the same experimental conditions.

We hypothesize that long-term application of biofertilizers in ‘Gala’ apple orchards improves soil fertility, enhances leaf nutrient status, and positively influences vegetative growth and yield compared to conventional mineral fertilization. This study aligns with the EU Farm to Fork strategy by quantitatively assessing nutrient use efficiency, soil fertility retention, and crop productivity under reduced mineral fertilizer regimes, providing measurable outcomes relevant to European sustainability targets.

Thus, the objectives of this study were to: (i) compare biofertilizer programs with conventional mineral fertilization regarding soil fertility, leaf nutrient status, vegetative growth, and yield and (ii) identify the most responsive indicators through multivariate analysis.

## 2. Results

### 2.1. Fruit Set Determination

Fruit set exhibited a clear temporal trend over the study period (Table 1), with the highest percentages observed in 2019, followed by declines in 2020 and 2021, and a slight recovery in 2022. Statistical analysis confirmed a significant effect of year on fruit set (F(3.608) = 28.81, *p* < 0.001), whereas fertilization treatment (F(7.608) = 1.78, *p* = 0.089) and the treatment × year interaction (F(21.608) = 1.13, *p* = 0.314) were not significant.

Although fruit set values varied numerically among treatments (e.g., 2019: 13.63 ± 3.52% for Myc2 to 30.47 ± 4.85% for Myc4; 2022: 7.72 ± 2.61% for Myc2 to 15.93 ± 2.86% for T100), no statistically significant differences were observed among treatments within the same year, consistent with the ANOVA results. Consequently, all treatments shared the same grouping letter (a) in each year (Games–Howell post hoc, *p* < 0.05).

### 2.2. Fruit Growth

**Figure 1:**
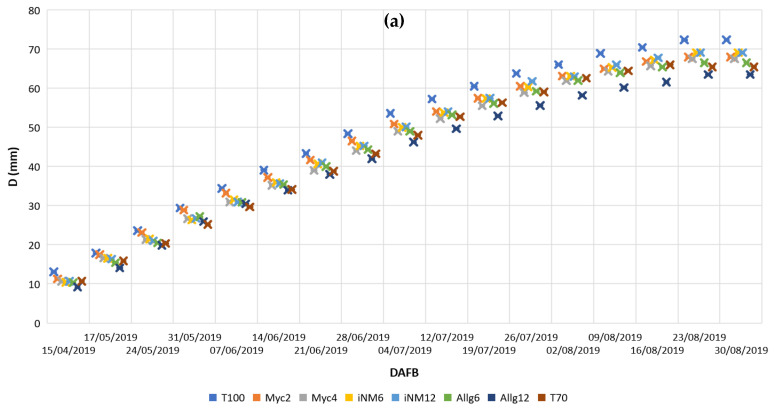

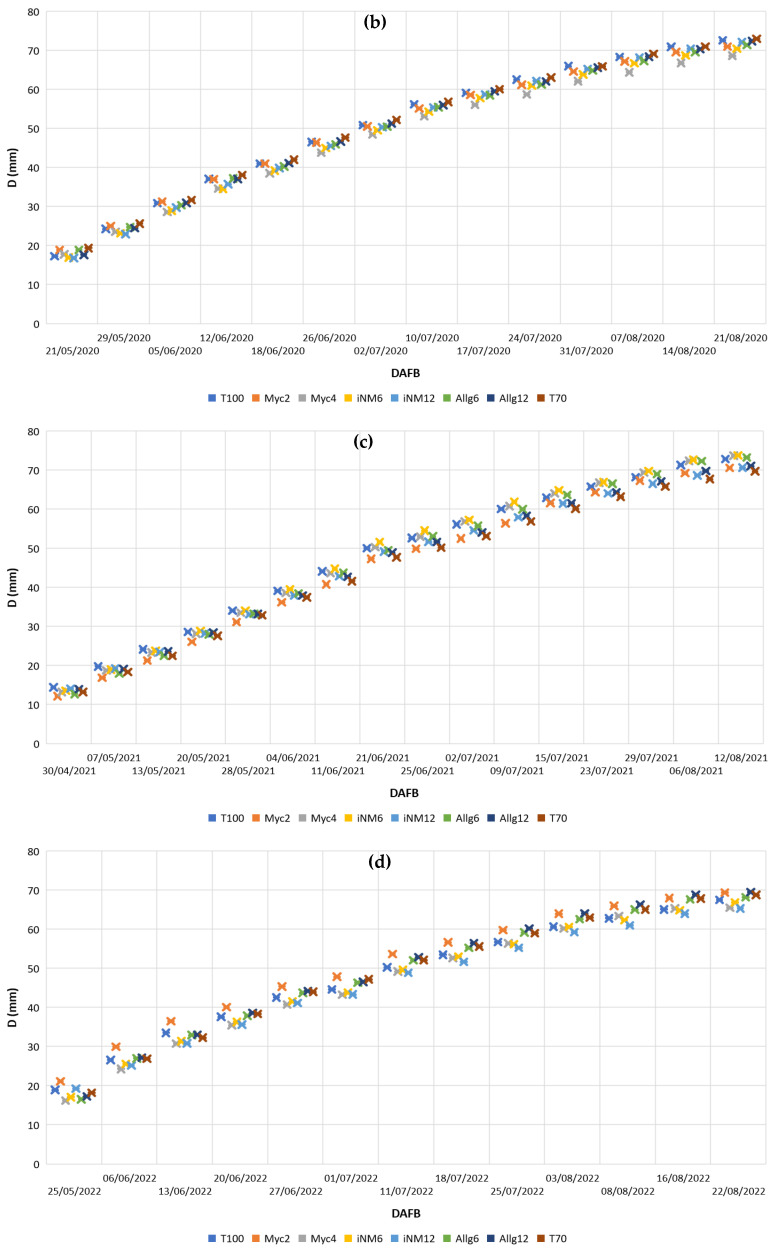
Weekly mean fruit diameter (D, mm), expressed in days after full bloom (DAFB), under different fertilization strategies during the experimental seasons: (**a**) 2019; (**b**) 2020; (**c**) 2021; and (**d**) 2022.

Fruit growth followed a sigmoidal pattern in all four seasons, with a rapid increase during the early stages (~0–50 days after full bloom, DAFB) and slower growth towards the end of the season (Figure 1).

In 2019 (Figure 1a), fruit measured diameters increased steadily from 13–72 mm across the season. T100, iNM12, and iNM6 exhibited the fastest early growth and reached the largest final diameters (72.35, 69.11, and 69.12 mm, respectively), while Myc4, Myc2, and Allg12 showed slower growth and smaller final sizes.

In 2020 (Figure 1b), all treatments displayed rapid growth up to ~50 DAFB, after which growth slowed. T70 and Myc2 produced the largest fruits (72.97 and 70.97 mm), whereas Myc4 and Allg12 had intermediate final diameters.

In 2021 (Figure 1c), fruit growth patterns were similar, with T100, Myc4, and iNM6 reaching the highest final diameters (72.86, 73.73, and 73.79 mm), while Allg6 and Myc2 remained smaller.

In 2022 (Figure 1d), Myc2 displayed the fastest growth during the early phase and achieved the largest final diameter (69.34 mm), followed by Myc4 and iNM6 (65.51 and 66.86 mm). Other treatments showed comparatively slower growth and smaller final sizes.

Overall, fruit growth dynamics were consistent across years, with early-season increases followed by a slower linear growth pattern. While mineral-based treatments often produced larger fruits, certain biofertilizer treatments (notably Myc2 in 2022) also achieved high final diameters, highlighting treatment-specific temporal differences in growth rates.

### 2.3. Fruit Production and Yield

Fruit production and yield showed clear temporal trends and treatment-specific responses over 2019–2022 (Table 2).

The number of fruits per tree increased from 2019 to 2022 in most treatments. In 2019, Myc2 (59.8 ± 4.72), Myc4 (55.2 ± 5.39), and Allg12 (59.5 ± 4.19) produced the highest numbers of fruits per tree, whereas Allg6 (37.4 ± 2.88) and iNM12 (37.2 ± 2.64) produced lower numbers. In 2020, T100 (44.8 ± 4.88) and T70 (39.6 ± 4.68) had the largest fruit numbers, while Myc4 (29.1 ± 4.17) and Allg12 (20.3 ± 2.83) had markedly lower numbers. During 2021–2022, T70, Myc4, and iNM6 consistently reached the highest fruit numbers, with T100, Myc2, and Allg12 showing intermediate values. Statistical analysis confirmed that year (F(3,288) = 66.96, *p* < 0.001), treatment (F(7,288) = 6.62, *p* < 0.001), and their interaction (F(21,288) = 2.48, *p* < 0.001) significantly affected fruit number.

Average fruit weight also varied among treatments and years. In 2019, T100 (145 ± 5.30 g) and iNM12 (151 ± 6.83 g) produced the heaviest fruits, whereas Myc2 (141 ± 3.34 g) and T70 (126 ± 6.65 g) produced lighter fruits. In 2020, fruit weight ranged from 121 ± 5.42 g (T100) to 158 ± 3.53 g (Allg6). In 2021, iNM6 (150 ± 5.97 g), Myc4 (138 ± 4.67 g), and Allg6 (146 ± 4.04 g) achieved the highest weights, while T70 (133 ± 3.97 g) and Myc2 (131 ± 5.99 g) achieved lighter weights. In 2022, the highest fruit weights were observed in T100 (137 ± 3.04 g) and Myc4 (144 ± 4.16 g). ANOVA confirmed that year (F(3,288) = 32.81, *p* < 0.001), treatment (F(7,288) = 3.71, *p* < 0.001), and their interaction (F(21,288) = 2.43, *p* < 0.001) were all significant.

Fruit production per tree reflected these patterns. In 2019, Allg12 (7.69 ± 0.45 kg/tree) and Myc4 (7.04 ± 0.54 kg/tree) achieved the highest values, while Allg6 (4.91 ± 0.37 kg/tree) and iNM12 (5.19 ± 0.33 kg/tree) had lower values. In 2020, T100 (6.27 ± 0.60 kg/tree) and T70 (5.68 ± 0.65 kg/tree) surpassed the other treatments. During 2021–2022, Myc4, T100, and iNM6 consistently produced the highest yields, with T100, T70, and Allg12 outperforming Myc2 and Allg6. ANOVA indicated that year (F(3,288) = 69.37, *p* < 0.001), treatment (F(7,288) = 6.07, *p* < 0.001), and the year × treatment interaction (F(21,288) = 1.93, *p* = 0.009) were significant.

Normalized production per hectare mirrored these patterns. Overall, fruit production dynamics highlighted both temporal increases and treatment-specific performance, with mineral-based and some allograft treatments frequently achieving higher fruit numbers and yields, while certain biofertilizer treatments (Myc2, Allg6) showed more moderate outputs.

**Figure 2:**
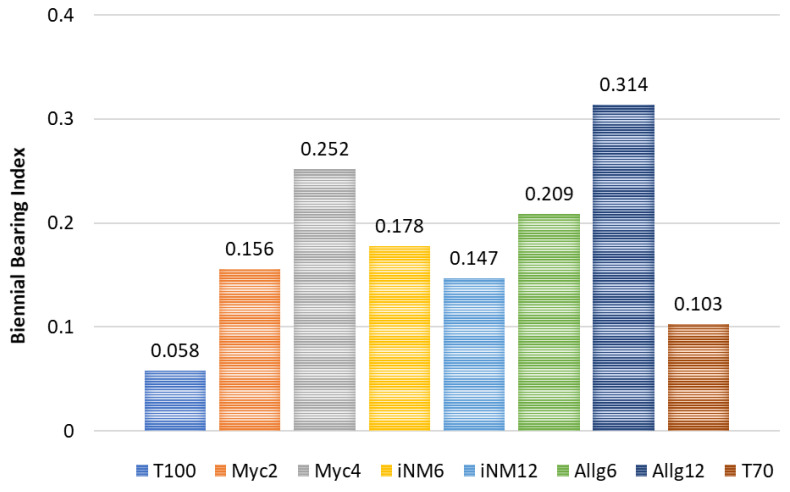
Biennial Bearing Index for apple trees under different fertilization treatments (2019–2022).

To evaluate the tendency for alternate bearing among treatments, the Biennial Bearing Index (BBI) was calculated based on the annual fruit production per tree. Figure 2 presents the BBI values for all treatments over the four-year period, highlighting differences in fruiting regularity. Treatments T100 and T70 exhibited the lowest BBI values, indicating more regular annual production, whereas Allg12 and Myc4 showed the highest BBI values, reflecting stronger alternation between years.

Pearson correlation analysis (Table 3) identified significant relationships among tree and fruit production parameters. The number of fruits per tree was negatively correlated with average fruit weight (r = –0.44, *p* < 0.001), indicating a trade-off between fruit quantity and individual size. Trunk cross-sectional area (TCSA) was strongly negatively correlated with yield per TCSA (r = –0.73, *p* < 0.001), suggesting that larger tree structures may reduce productivity at the shoot level.

### 2.4. Fruit Quality

Fruit quality parameters, including diameter, weight, firmness, Brix, hue, and dry matter, varied over the four-year study period and among fertilization treatments (Table 4). Year-to-year differences were also evaluated for each treatment (see F and *p* values above).

Fruit diameter (mm) showed significant variation across years (F(3.768) = 177.52, *p* < 0.001) and a significant year × treatment interaction (F(21.768) = 2.46, *p* < 0.001), whereas treatment alone was not significant (F(7.768) = 0.34, *p* = 0.937). Diameters peaked in 2020 for Myc2 (73.53 ± 0.73 mm) and iNM6 (72.86 ± 0.70 mm) and declined by 2022, with the lowest values observed in Myc2 and iNM6 (66.99 ± 0.33 and 66.99 ± 0.22 mm, respectively).

Average fruit weight (g) varied significantly with year (F(3.768) = 110.15, *p* < 0.001) and year × treatment interaction (F(21.768) = 2.67, *p* < 0.001), but not with treatment alone (F(7.768) = 1.16, *p* = 0.321). Maximum weights were observed in 2020 for Myc2 (163.58 ± 5.34 g) and iNM6 (162.82 ± 4.28 g), declining across all treatments by 2022, particularly for Allg12 (127.18 ± 1.50 g), Myc2 (129.10 ± 3.11 g), and iNM6 (128.39 ± 1.58 g).

Fruit firmness (kg/cm^2^) was affected by year (F(3.768) = 21.24, *p* < 0.001) and year × treatment interaction (F(21.768) = 2.98, *p* < 0.001), with no significant effect of treatment (F(7.768) = 0.13, *p* = 0.996). Firmness remained comparable across all fertilization modalities throughout 2019–2022, with minor seasonal fluctuations. Values ranged from 7.38 ± 0.23 kg/cm^2^ (Allg12, 2020) to 9.36 ± 0.18 kg/cm^2^ (T70, 2019).

Brix (°) varied significantly with year (F(3.768) = 38.25, *p* < 0.001), treatment (F(7.768) = 4.15, *p* < 0.001), and their interaction (F(21.768) = 2.38, *p* < 0.001). The highest sugar content occurred in 2020–2021 for Allg12 (15.92 ± 0.26 °Brix) and iNM6 (15.18 ± 0.25 °Brix), while lower values were observed in 2019. By 2022, Brix values stabilized across treatments (range 13.90 ± 0.25 to 18.67 ± 0.24 °Brix).

Hue (°) was influenced by year, treatment, and their interaction (*p* < 0.01). Maximum mean hue was recorded in 2021 (38.45 ± 2.25, Allg12) and minimum in 2019 (25.96 ± 0.58). Myc2 and T100 generally showed higher hue values, whereas Allg12 and Allg6 showed lower values.

Dry matter (%) increased significantly with year (*p* < 0.001) and treatment (*p* = 0.015), while the year × treatment interaction was not significant (*p* = 0.148). Highest values occurred in 2022 (18.17 ± 0.26 °, T100), with Myc2 and iNM6 consistently showing higher dry matter content.

Overall, fruit quality showed clear temporal dynamics, with peaks in diameter and weight in 2020, increased hue in 2021, and the highest dry matter in 2022. Treatments iNM6, Myc2, and Allg12 frequently achieved superior quality metrics, while Allg6 and some other treatments performed less consistently.

### 2.5. Fruit Size Caliber

**Figure 3:**
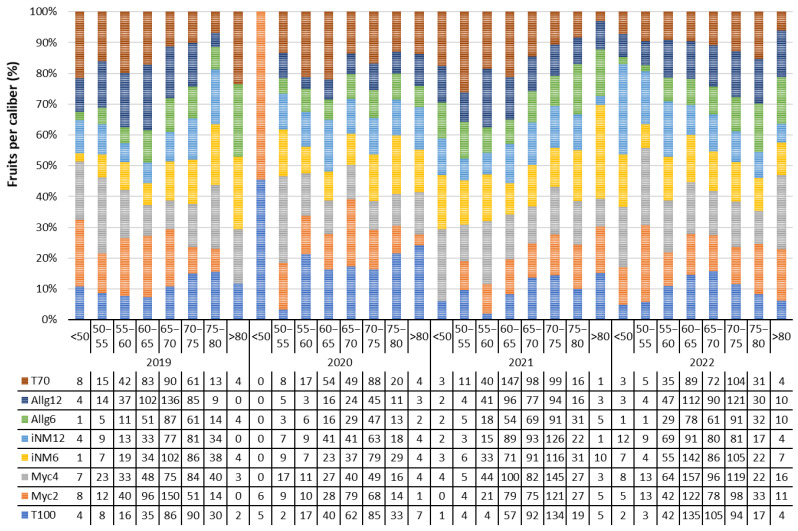
Distribution of apple fruit numbers across different fruit calibers (<50, 50–55, 55–60, 60–65, 65–70, 70–75, 75–80, and >80 mm) for each treatment during the experimental seasons. Bars are stacked to 100% to illustrate the relative contribution of each treatment within each caliber class.

Fruit size distribution (<50, 50–55, 55–60, 60–65, 65–70, 70–75, 75–80, and >80 mm) was assessed for each fertilization treatment from 2019 to 2022 (Figure 3).

No significant differences among treatments were detected (Kruskal–Wallis χ^2^_7_ = 1.314, *p* = 0.988; Dunn post hoc Bonferroni *p* > 0.05), indicating that fertilization did not markedly alter the overall size distribution.

Across all years, medium-sized fruits (60–75 mm) predominated. Fruits larger than 65 mm, which represent the highest commercial value, were consistently produced in greater numbers by T100, Myc2, Myc4, iNM6, and iNM12, whereas Allg6, Allg12, and T70 yielded fewer high-value fruits.

Temporally, the proportion of larger fruits (>65 mm) increased from 2019 to 2022 for most treatments, particularly T100, Myc4, and iNM6, which maintained high numbers of fruits in the 65–75 mm range. Smaller fruits (<60 mm) were generally scarce, especially in 2021 and 2022.

Overall, the data indicate that while fertilization treatments did not significantly change the overall fruit size distribution, certain treatments consistently enhanced the production of commercially desirable, large-caliber fruits over the four years.

### 2.6. Soil Chemical and Microbiological Parameters

**Figure 4:**
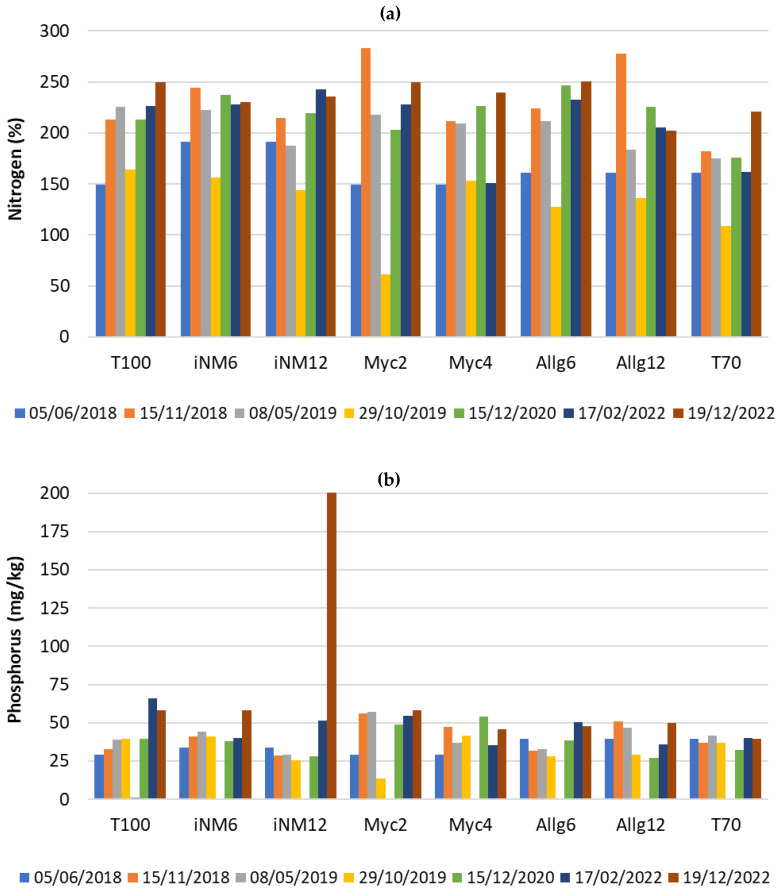

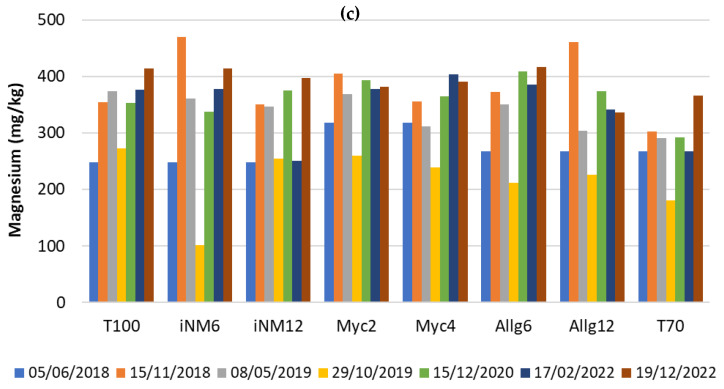
Key soil chemical parameters from 2018 to 2022 across all treatments: (**a**) total nitrogen (N, %); (**b**) phosphorus (P, mg/kg); and (**c**) magnesium (Mg, mg/kg).

Figure 4 illustrates the temporal evolution of key soil chemical parameters—total nitrogen (N), available phosphorus (P), and magnesium (Mg)—across treatments from 2018 to 2022. These parameters were selected due to their major role in soil fertility and apple tree nutrition.

Total nitrogen (Figure 4a) displayed moderate variability over the monitoring period. N concentrations in all treatments were relatively low in the initial 2018 sampling (0.09%), followed by a general increase by May 2019, particularly in T100, Myc2, Allg12, and T70. After this peak, N values tended to return to baseline levels and remained relatively stable through 2020–2022, suggesting a transient response to fertilization and mineralization events rather than a long-term accumulation.

Available P (Figure 4b) showed pronounced temporal fluctuations, reflecting fertilizer application and soil P dynamics. The first peak occurred in November 2018 in iNM6 and iNM12 (139 and 117 mg/kg, respectively), while Allg12 exhibited a maximum of 126 mg/kg in the same sampling. A high value was observed in December 2022 for iNM12 (1307 mg/kg); however, given that soil samples were composited, this point had minimal influence on the overall mean. Therefore, it likely reflects natural variability rather than a localized fertilizer residue or soil heterogeneity. Apart from this extreme value, most treatments converged to moderate P levels (80–145 mg/kg) by the final sampling.

Mg concentrations (Figure 4c) remained generally stable over time but tended to be higher in 2022 compared with early samplings in several treatments, particularly T100 (414 mg/kg), iNM6 (414 mg/kg), and Allg6 (416 mg/kg). These increases may indicate gradual release from the soil exchange complex and/or the cumulative effect of repeated biofertilizer or mineral fertilizer applications.

Overall, these results suggest that soil macronutrient availability in this orchard is dynamic and strongly influenced by fertilization events, with only Mg showing a tendency for long-term buildup.

Prior to PCA, data suitability was evaluated using the Kaiser–Meyer–Olkin (KMO) measure of sampling adequacy (overall MSA = 0.467) and Bartlett’s Test of Sphericity (χ^2^ = 706.478, df = 120, *p* < 0.001), confirming that the dataset was appropriate for multivariate analysis.

Rotated Principal Component Analysis (PCA) of standardized soil parameters identified four main components explaining 65.9% of the total variance (Table 5). RC1 (18.7% of variance) was primarily associated with sulfur (S), electrical conductivity (EC), boron (B), iron (Fe), and Mg, reflecting overall soil mineral load and salinity. RC2 (17.4%) showed very high positive loadings for soil pH (H_2_O and CaCl_2_) and moderate loading for Ca, representing a pH control axis. RC3 (16.2%) grouped zinc (Zn) and potassium (K) positively, but organic matter (OM) and cation exchange capacity (CEC) negatively, suggesting a trade-off between available K/Zn and soil buffering capacity. RC4 (13.6%) was dominated by total nitrogen (N, positive) and C/N ratio (negative), together with available P (positive), representing a nitrogen–phosphorus fertility axis. Overall, the PCA highlights that biofertilizer programs shift the multivariate nutrient profile of the soil rather than acting on single parameters in isolation.

To assess whether treatments differed significantly along the multivariate gradients identified by the PCA, scores of the four retained components (PC1–PC4) were compared among treatments using one-way ANOVA. The mean scores ± standard deviations for each treatment are shown in Table 6. Significant differences were detected only for T100 in PC3 and PC4 (*p* < 0.05), while no significant effects were observed for PC1, PC2, or any other treatment (ns).

Cumulative changes from 2018 to 2022 (Table 7) revealed clear treatment-specific trends in soil chemical properties.

Soil pH (H_2_O and CaCl_2_) increased in all treatments (≈19–25%), suggesting gradual neutralization or liming effects over time, with the largest relative increases in T100, Myc2, and Myc4. EC increased sharply in iNM6 (+80%) and iNM12 (+56%), indicating a higher accumulation of soluble salts under mineral fertilization. In contrast, EC remained almost unchanged in Allg12 (+2%). OM generally declined across treatments (−2% to −28%), particularly under iNM6 (−28%) and Allg12 (−25%), suggesting enhanced mineralization or lower residue input. N showed mixed responses: in T100 it increased (+33%) while in other treatments it showed small declines (−9% to −23%). The C:N ratio decreased consistently, except in Allg6, Allg12, and T70), reflecting faster organic matter turnover and relatively higher N availability. Available P increased markedly in iNM12 (+1445%, which may reflect an outlier, as mentioned above) and in Myc2 (+101%) and T100 (+99%). Potassium also increased in all treatments (18–94%), with T100 showing the highest gain.

From 2018 to 2022, nutrient dynamics were treatment-dependent. Ca and Mg increased in all treatments, with the highest rises under T100 and Myc2 (+43–67%). Sulfur (S) showed contrasting trends: it surged under iNM6 (+261%) and iNM12 (+198%) but declined under Myc4 (−48%), Allg6 (−14%), Allg12 (−23%), and T70 (−29%). Among micronutrients, B and Zn generally increased (B up to +47% in iNM12; Zn up to +413% in INM12), while Cu exhibited modest rises and Fe mostly declined. CEC decreased in nearly all treatments, most strongly in iNM6 (−21%) and Myc2, Myc4, Allg6 and Allg12 (−19%), likely reflecting reduced organic matter contribution. Overall, mineral fertilization (iNM6 and iNM12) strongly enhanced nutrient availability, particularly S, P, and Cu, whereas biofertilizers induced moderate changes, maintaining balanced micronutrients but sometimes leading to S and OM losses.

Microbiological analyses, limited to two sampling events conducted by the company Soilvitae, Lda., were evaluated for reference only. In June 2019, total culturable bacteria did not differ among treatments. iNM6 and Myc2/Myc4 increased densities of phosphorus-solubilizing and -mineralizing bacteria compared with the T70. Nitrogen-fixing bacteria were higher in iNM12 and Allg6 than in all controls. Dehydrogenase activity was elevated in iNM6 and Myc2/Myc4 compared with the 70% control. Extracellular enzymes—including β-xylosidase, *N*-acetylglucosaminidase, β-glucosidase, and phosphatases—showed higher activity in biofertilizer treatments relative to the 70% control. Mycorrhizal colonization was also higher in Myc2/Myc4-treated soils, with arbuscules and vesicles more abundant at higher tablet doses.

In July 2020, total culturable bacteria remained around 10^6^ CFU/g, slightly lower in Myc2/Myc4 treatments. Phosphorus-solubilizing and -mineralizing bacteria showed no significant differences, although iNM6 and Allg6 tended to have higher mineralizing populations. Nitrogen-fixing bacteria were higher in Allg6 than in the 100% control, but similar to the 70% control. Dehydrogenase activity showed no significant differences among treatments, though overall activity was higher than in the previous year. Extracellular enzyme activity remained elevated in certain biofertilizer treatments and in the 70% control. Mycorrhizal colonization remained higher in Myc2/Myc4-treated soils, without statistically significant differences.

### 2.7. Leaf Nutrient Parameters

**Figure 5:**
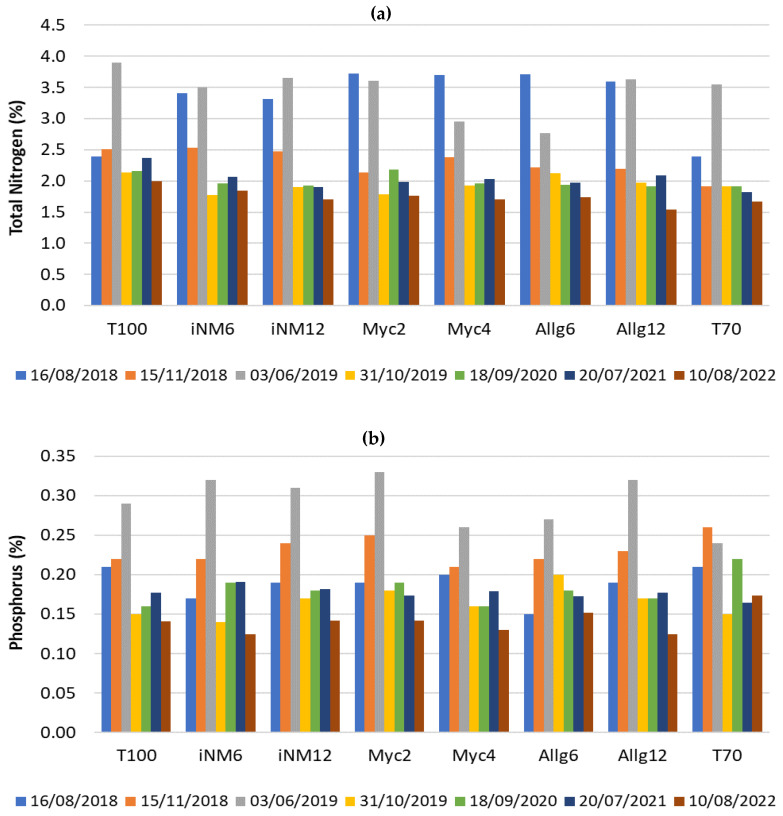

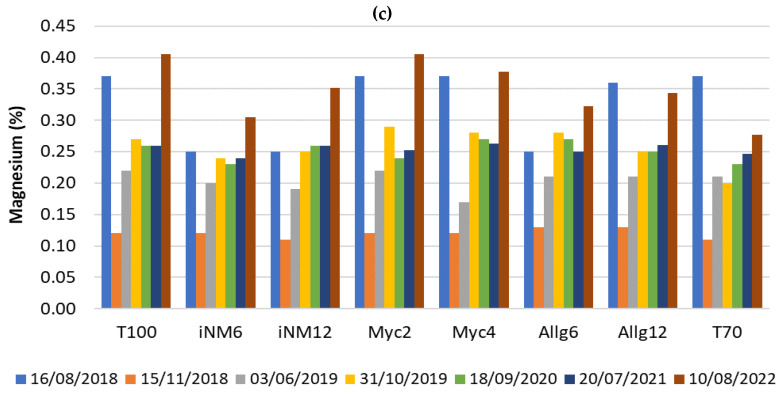
Key leaf nutrients from 2018 to 2022 across all treatment: (**a**) total nitrogen (N, %); (**b**) phosphorus (P, %); and (**c**) magnesium (Mg, %).

Figure 5 shows the temporal evolution of leaf nitrogen (N), phosphorus (P), and magnesium (Mg) concentrations across treatments from 2018 to 2022. These nutrients were chosen due to their relevance for photosynthetic function and overall tree performance. Concentrations are expressed in elemental form (% N, % P, and % Mg), which differs from soil reporting (e.g., P and Mg).

Leaf N (Figure 5a) started relatively high in 2018 (≈2.4–3.7%), reached a peak in June 2019 in most treatments (notably T100 with 3.9%), and gradually declined in subsequent years, stabilizing between 1.5–2.0% by 2022. Treatments with inoculants (iNM6 and iNM12) and mycorrhizae (Myc2 and Myc4) generally maintained higher N values than T70 (control) after 2019. Leaf P (Figure 5b) showed modest short-term increases in 2019, with iNM6, iNM12, Allg12 and Myc2 reaching ≈0.31–0.33%, compared with lower values in T70 (0.24%). After 2020, P concentrations converged among treatments, remaining near 0.13–0.17%. Leaf Mg (Figure 5c) displayed more variable dynamics: concentrations rose in 2019 in T100, Myc2, and Myc4 (≈0.22–0.29%), declined slightly in 2020–2021, and peaked again in 2022, particularly in T100 (0.41%), Myc2 (0.41%) and Myc4 (0.38%).

Overall, these patterns reflect nutrient pulses following fertilization events, followed by a stabilization phase, with some treatments maintaining a nutritional advantage in the later years.

Rotated PCA of standardized leaf nutrient concentrations identified three main components explaining 72.2% of the total variance (Table 8). Sample adequacy was assessed using the KMO test, with an overall MSA of 0.65, indicating moderate suitability for RCA, and Bartlett’s test of sphericity was significant (χ^2^ = 709.791, df = 91, *p* < 0.001), confirming that correlations among variables were sufficiently strong for analysis.

RC1 (35.8% of variance) showed strong positive loadings for Zn, P, N, and B and negative loadings for Fe and Al, reflecting a micronutrient–P fertility axis. RC2 (18.6%) was mainly driven by Mn (positive) and Ca (negative), capturing cation balance and Ca–Mn antagonism. Total N also had a moderate loading on RC2 (0.582), indicating some overlap between components. PC3 (17.8%) was strongly associated with Mg and Ca (positive) and K (moderate positive) and negatively with Na and S, representing a cation–salinity gradient. Overall, the PCA indicates that biofertilizer treatments coordinated shifts in leaf composition, particularly enhancing Zn–P–N status (RC1) and improving Mg–K balance while reducing Na accumulation (RC3).

To assess whether treatments significantly influenced these multivariate patterns, scores of the three retained components (PC1–PC3) were compared among treatments using one-way ANOVA. The mean scores ± standard deviations for each treatment are shown in Table 9. No significant differences were detected for any component or treatment (ns), suggesting that leaf nutrient profiles remained relatively stable across the fertilization regimes.

Over the study period, leaf nutrient dynamics varied substantially depending on treatment (Table 10). N and P consistently declined across treatments (−16% to −57% for N; −50% for P in most), whereas K increased in all plots (13–100%). Mg remained relatively stable (0% change in most treatments), while Ca showed high increases, particularly in Myc2/Myc4 (+100–89%) and Allg6 (+88%). S decreased in all treatments (−50% to −67%), and Mn exhibited strong declines across all treatments (−79% to −91%). In contrast, Fe accumulated substantially (119–210%), especially under biofertilizer treatments. Among micronutrients, B showed minor or inconsistent changes (−7% to −31%), Cu varied with treatment, with notable increases under Myc2 (+76%), and Zn decreased in all treatments. Na and Al showed pronounced accumulation in almost all treatments (Na +101% to +500%; Al +205% to +470%), indicating progressive enrichment. Molybdenum (Mo) was negative in all treatments, except in T70 (+33%). Biofertilizer treatments generally promoted accumulation of Fe and Al, with substantial gains in K under some plots and variable effects on Na, while Mg levels were mostly maintained under biofertilizers, and mineral fertilization (T100 and T70) showed stable Mg in T100 but a moderate decline in T70, providing a comprehensive view of how leaf nutrient status evolved under different soil management strategies.

### 2.8. Tree Growth and Biometric Assessment

Tree growth over the 2018–2022 period showed clear treatment-specific patterns (Table 11).

Tree height (TH) differed among treatments in each year (Games–Howell post hoc, *p* < 0.05). In 2018, trees in Myc2 (2.16 ± 0.03 ^b^) and Myc4 (2.10 ± 0.04 ^a^^b^) were slightly taller than in other treatments, while those in Allg6 (2.13 ± 0.02 ^b^), Allg12 (2.09 ± 0.04 ^a^^b^), iNM12 (2.01 ± 0.04 ^a^^b^), iNM6 (2.06 ± 0.04 ^a^^b^), T100 (1.95 ± 0.07 ^a^) and T70 (2.13 ± 0.02 ^b^) were similar. Heights remained generally similar among treatments in subsequent years, with minor fluctuations (e.g., 2022: Myc4 2.86 ± 0.06 ^a^, iNM6 2.80 ± 0.04 ^a^, Myc2 2.72 ± 0.02 ^a^, T100 2.74 ± 0.05 ^a^, T70 2.74 ± 0.03 ^a^).

Canopy volume (CV), measured only in 2020–2022, was highest in Myc4 in 2020 (2.21 ± 0.12^c^) and intermediate in iNM6 (1.86 ± 0.07 ^a^^b^), whereas differences among treatments were smaller in 2021 and 2022, with all treatments statistically similar.

TCSA differed among treatments in all years. In 2018, higher values were observed in Myc4 (3.00 ± 0.23 ^b^^c^) and Myc2 (3.09 ± 0.25 ^c^), while values for T100 (2.09 ± 0.18 ^a^), iNM6 (2.36 ± 0.15 ^a^^b^^c^), iNM12 (2.26 ± 0.17 ^a^^b^), Allg6 (2.13 ± 0.13 ^a^) and Allg12 (2.54 ± 0.16 ^a^^b^^c^) were lower. From 2020 onwards, TCSA increased in all treatments, but differences were less pronounced (2022: 9.15–11.91 cm^2^).

Shoot growth varied significantly among treatments each year. Myc4, Myc2, and iNM6 generally showed the largest annual increments (2022: Myc4 30.43 ± 3.55 ^a^, iNM6 28.57 ± 4.03 ^a^, Myc2 23.60 ± 1.00 ^a^), whereas T100 and T70 consistently had lower annual increments (2022: T100 19.32 ± 2.15 ^a^, T70 22.45 ± 2.55 ^a^). Consequently, cumulative shoot growth by 2022 was highest in Myc4 (68.79 m), iNM6 (60.25 m), and Myc2 (52.15 m), and lowest in T100 (44.68 m) and T70 (48.97 m).

**Figure 6:**
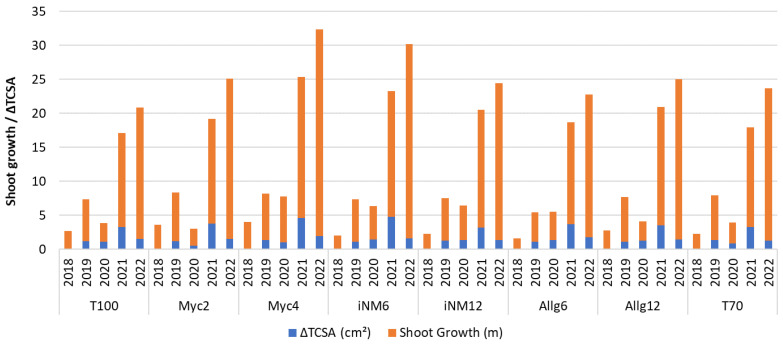
Annual shoot growth and annual trunk cross-sectional area increment (∆TCSA) for each treatment from 2018 to 2022. Shoot growth (m) represents the annual elongation of the shoots, while ∆TCSA (cm^2^) represents the yearly increase in trunk cross-sectional area relative to the previous year.

Annual shoot growth and annual trunk cross-sectional area increment (∆TCSA) showed clear temporal patterns (Figure 6). Shoot growth increased steadily from 2018 to 2022 for all treatments, with Myc4, iNM6, and Allg12 consistently exhibiting the highest annual increments. For instance, in 2022, Myc4 reached 30.43 m, iNM6 28.57 m, and Allg12 23.52 m, while conventional treatments T100 and T70 recorded lower values (19.32 m and 22.45 m, respectively).

∆TCSA also increased over time, particularly after 2020, reflecting accelerated trunk thickening. Myc4 and iNM6 consistently showed the largest annual increments, peaking at 4.57 cm^2^ (Myc4, 2021) and 4.79 cm^2^ (iNM6, 2021). Myc2, iNM12, and Allg12 had intermediate ∆TCSA, whereas T100 and T70 exhibited the smallest increases throughout the study period.

These trends indicate that treatments involving microbial inoculants (Myc and iNM) promoted both shoot elongation and trunk growth more effectively than conventional fertilization.

## 3. Discussion

### 3.1. Fruit Growth, Yield and Quality

Fruit development, yield, and quality were strongly influenced by climatic conditions, with fertilization modulating these effects. Inter-annual variation in spring temperatures and solar radiation affected flowering, pollen viability, stigma receptivity, and early fruit retention [29,30]. Cooler or highly variable bloom temperatures reduced fruit set, whereas moderate temperatures and solar radiation favored early retention.

Fertilization had minor direct effects on fruit set but could indirectly influence retention via tree vigor and canopy microclimate [31,32]. Early fruit growth followed compensatory dynamics consistent with source–sink theory: a lower initial fruit set enhanced assimilate availability per fruit, resulting in larger diameters [33,34,35].

Average fruit diameter and weight showed year × treatment interactions, with highest values in iNM12, T100, Myc2, and iNM6 during 2019–2020, suggesting that nutrient availability modulated growth under favorable climate, though climate remained the predominant driver [36,37].

Fruit firmness, soluble solids (Brix), hue, and dry matter content varied across years, reflecting complex interactions between fertilization and environment [38,39,40]. Medium-sized fruits (60–75 mm) predominated across all treatments, while larger fruits (>65 mm) were more frequent in T100, Myc2, Myc4, iNM6, and iNM12, again showing the primary influence of climate.

Correlations showed that larger TCSA was associated with lower yield per shoot (r = −0.73, *p* < 0.001), supporting the trade-off between vegetative growth and fruit production [41,42,43]. Overall, fertilization strategies modulate fruit growth, yield, and quality, but seasonal climatic conditions dominate, emphasizing the need for adaptive management that accounts for nutrient application, crop load, and environmental monitoring [44].

Alternate bearing tendency, as assessed by the Biennial Bearing Index (BBI), varied among treatments, reflecting differences in fruiting regularity. Treatments T100 and T70 showed the lowest BBI values, indicating more consistent annual fruit production, whereas Allg12 and Myc4 exhibited higher BBI values, suggesting stronger alternation between years. These results highlight that balanced fertilization strategies can help mitigate alternate bearing, although climatic conditions and inter-annual variability remain the key drivers of production stability [45,46].

### 3.2. Soil Chemical and Microbiological Dynamics

Over the five-year study (2018–2022), soil chemical dynamics appeared to be influenced by fertilization strategy, biofertilizer application, and climatic variability. Given the limited temporal and spatial replication, these observations should be interpreted cautiously, but they allow the formulation of plausible hypotheses regarding nutrient behavior in the orchard.

Increases in soil pH observed in treatments such as T100, Myc2, Myc4, iNM6, and iNM12 may reflect the combined effects of fertilization inputs and the natural buffering capacity of the soil during drier periods, consistent with reports in temperate orchards [47,48,49]. We hypothesize that repeated applications of mineral fertilizers and biofertilizers can modulate pH locally, potentially influencing nutrient availability and microbial activity.

Cumulative increases in EC, particularly in biofertilizer-treated plots, could result from the combination of repeated nutrient inputs and high summer evapotranspiration. This pattern suggests that biofertilizers may enhance nutrient solubilization and uptake, while also highlighting the need for integrated nutrient management to prevent potential salinization under Mediterranean conditions [50].

Observed declines in organic matter in some treatments, alongside increases in total nitrogen in others, may indicate differential nutrient turnover dynamics. We propose that microbial inoculants, especially those containing *Glomus* spp. or *Bacillus* spp., could accelerate organic matter mineralization, enhancing short-term nutrient availability. Reductions in C:N ratio could similarly reflect increased microbial decomposition activity; however, the limited microbiological dataset prevents formal statistical confirmation [51].

Macronutrient trends were variable. Notable increases in K (up to +94% in T100), Mg (up to +67% in T100/Myc2), and P (up to +1445% in iNM12, though likely an outlier) under specific biofertilizer and mineral fertilizer treatments suggest potential enhancement of nutrient stocks, possibly due to improved nutrient solubilization and uptake mediated by microbial activity. Extreme values, such as high P in iNM12 (elemental form, 1307 mg/kg), might reflect site-specific soil conditions interacting with fertilization. The observed increase in K relative to T70 may be linked to the presence of *Bacillus* spp. in Kiplant formulations or the benefits of *Glomus* spp. in Mycoshell^®^ applications, but these remain tentative hypotheses in the absence of extensive replication.

Micronutrient fluctuations and decreases in CEC suggest complex interactions among fertilization, soil chemistry, and climate, potentially mediated by microbial processes. Microbiological data from 2019 and 2020, although limited, indicate that biofertilizers could enhance populations of phosphorus-solubilizing and -mineralizing bacteria, nitrogen-fixing organisms, and mycorrhizal colonization, particularly in Mycoshell^®^ treatments [11,12,13,52,53,54]. These patterns support the hypothesis that microbial inoculants can contribute to nutrient cycling, even if temporal dynamics cannot be formally established.

Rotated PCA of soil parameters, using elemental forms for P, K, Mg, and Ca, followed by ANOVA on the component scores, indicated that main treatment effects were captured in PC3 and PC4, with T100 showing significant differences, whereas other components and treatments were not statistically distinct. Overall, these results suggest that soil macronutrient availability in this orchard is dynamic and influenced by fertilization timing, nutrient mobility, and microbial activity, with Mg showing potential for gradual accumulation under repeated inputs, although formal confirmation is limited by the experimental design [55,56,57].

### 3.3. Leaf Nutrient Dynamics

Leaf nutrient concentrations exhibited temporal and treatment-dependent patterns influenced by fertilization and climatic conditions. Total nitrogen generally declined in most treatments, whereas conventional fertilization plots maintained relatively higher levels, suggesting more stable N availability. We hypothesize that differences in leaf N reflect both the timing of fertilizer application and the capacity of microbial inoculants to enhance short-term nutrient uptake [58].

Early-season peaks in N coincided with favorable temperature and rainfall, potentially facilitating nutrient absorption [59,60]. Phosphorus trends mirrored N, with transient increases post-fertilization followed by convergence across treatments. Using elemental P, leaf concentrations stabilized at 0.13–0.17% by 2022, with early peaks in biofertilizer and mineral fertilizer plots reaching 0.31–0.33%, reflecting both uptake and redistribution within the tree [61].

Magnesium and potassium showed selective accumulation in some biofertilizer treatments, with Mg peaks up to 0.41% in T100/Myc2, reflecting elemental form, suggesting possible synergistic effects between microbial activity, soil moisture, and nutrient availability [62]. Calcium remained relatively stable, while sulfur generally declined, reflecting differential mobility and plant demand.

Micronutrients demonstrated variable responses, with Zn and Cu tending to accumulate in biofertilizer-amended plots and Fe generally increasing (up to +210%), consistent with elemental reporting. Na and Al showed pronounced accumulation (+101% to +500% for Na; +205% to +470% for Al), reflecting progressive enrichment under biofertilizers, while Mo remained negative except in T70 (+33%).

Rotated PCA of leaf nutrient parameters, followed by ANOVA on the component scores, revealed no significant differences among treatments (ns), suggesting that multivariate leaf composition remained relatively stable even after adjusting to elemental forms. Overall, these patterns support the tentative hypothesis that biofertilizers may enhance uptake of certain cations and micronutrients (e.g., Zn, K, and Fe), while conventional fertilization better maintains N status under fluctuating environmental conditions [63,64,65,66].

### 3.4. Tree Growth and Biometric Performance

Apple tree growth and biometric parameters were influenced by both fertilization treatment and year. Trees receiving microbial inoculants (Myc4, Myc2, and iNM6) often exhibited greater height, canopy volume, trunk cross-sectional area, and shoot growth compared to conventional fertilization, suggesting potential benefits of biofertilizers for vegetative development, although climatic variability likely contributed to observed differences [67,68,69].

The observed superior performance of *Glomus* spp.-containing biofertilizers (Mycoshell^®^) may be attributed to their high endomycorrhizal content, which enhances early root growth, improves nutrient (N, P, and K) and water uptake, and accelerates vegetative development. Additionally, the timing of application at planting ensures early colonization and synergistic interactions with soil nutrients, potentially explaining the greater increases in trunk cross-sectional area, canopy volume, and shoot growth relative to other biofertilizers.

Peak shoot elongation and TCSA increments during 2021–2022 coincided with warmer spring–summer periods and moderate rainfall, reinforcing the hypothesis that climate strongly modulates growth dynamics [70,71].

Within the living mulch system, microbial inoculant treatments generally outperformed conventional fertilization, suggesting potential synergistic effects between soil organic matter retention, microbial activity, and nutrient availability [72,73]. The absence of significant treatment × year interactions for TCSA indicates relatively consistent vegetative responses, although limited replication warrants cautious interpretation [74,75].

Overall, these observations support the cautious hypothesis that integrating microbial inoculants with sustainable soil management can improve tree structure and vegetative performance, while acknowledging that climate remains a key modulator of growth responses.

## 4. Materials and Methods

### 4.1. Experimental Site and Collaborative Framework

**Figure 7:**
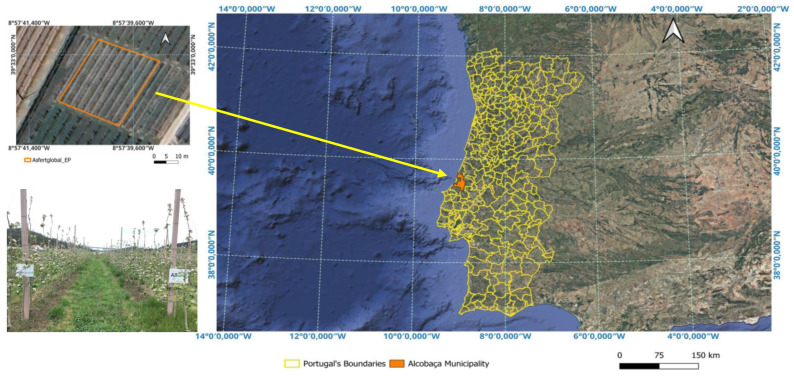
Location of the experimental orchard in Alcobaça, Portugal. National boundaries are shown in yellow; Alcobaça municipality is shaded in orange. Top left inset: detailed orchard view (orange outline; Google Earth, July 2025). Bottom left inset: orchard photograph (18 May 2021).

This study was conducted in a pilot experimental drip-irrigated apple orchard planted in May 2018 with branched trees of the *Malus domestica* Borkh ‘Gala Redlum’ cultivar, grafted onto M9 rootstock, at a planting density of 3472 trees ha^−1^ (spacing: 3.20 m × 0.90 m). The experimental plot covered 922 m^2^ (0.092 ha) and is located in Alcobaça, central-western Portugal, a transitional region influenced by both Atlantic and mountainous climatic factors (Figure 7). The orchard was established as a pilot trial on previously fallow land (5 years), minimizing pre-existing variability in soil nutrients. The study covered the 2018–2022 growing seasons. The experimental methodology was progressively refined over time: some parameters were only introduced in the second or third year of monitoring, and the number of samples for certain measurements increased in subsequent years. This adaptive approach allowed the protocol to better capture orchard development and the dynamics of soil–plant interactions.

The study was conducted under a collaborative protocol with AsfertGlobal, which provided financial support and biofertilizer products. All field management, fertilization, data collection, and analysis were performed independently by INIAV researchers, ensuring scientific integrity. Laboratory analyses were conducted by an independently accredited laboratory.

The region has a Mediterranean climate (Csb, Köppen–Geiger) with mild, wet winters and dry summers [76]. Average annual precipitation is 696 mm, mostly from October to March; mean annual temperature is 15.4 °C (max: 18.9 °C, min: 12.5 °C) [77].

The soil is clayey, with low organic matter (<2%) and pH (H_2_O) ranging from 6.2 to 8.0. A spontaneous herbaceous cover was maintained between rows, and herbicides were applied in a 1 m wide strip along tree lines until the beginning of flowering and during the month of November. Chemical fruit thinning was performed to ensure uniform crop load. Trees were trained in a central leader system, with pruning and pest/disease management conducted according to integrated fruit production standards and local commercial practices. Irrigation was supplied via a drip system (4 L h^−1^ per emitter, 0.9 m spacing) and controlled with soil sensors to maintain optimal water status (Irristrat^®^, Hidrosoph, Oeiras, Portugal).

### 4.2. Meteorological Data

**Figure 8:**
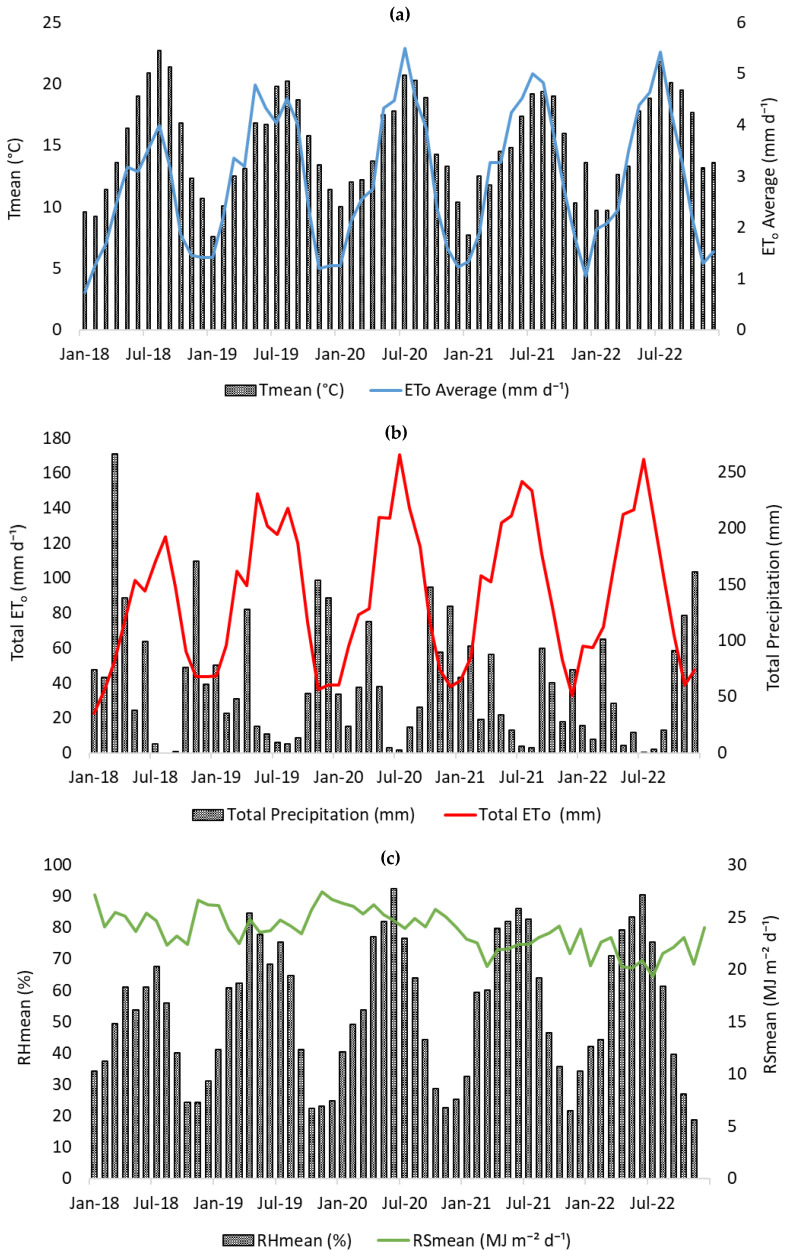
Climatic variables from 2018 to 2022: (**a**) daily mean air temperature (Tmean, °C) and reference evapotranspiration (ET_o_, mm d^−1^); (**b**) monthly totals of ET_o_ (mm) and precipitation (mm); and (**c**) daily mean relative humidity (RH, %) and solar radiation (RS, MJ m^−2^ d^−1^).

Figure 8 presents the climatic variables from 2018 to 2022. Figure 8a shows the mean air temperature (Tmean) and daily reference evapotranspiration (ET_o_) from 2018 to 2022. Both variables followed a typical seasonal trend, peaking during July–August, in accordance with the *Csb* Köppen–Geiger scale. The warmest year was 2022, with a maximum Tmean of 21.8 °C in July, whereas 2019 had the lowest winter temperatures (7.6 °C in January). These interannual fluctuations reflect varying atmospheric water demand that can influence apple tree phenology and irrigation requirements.

Monthly precipitation (Figure 8b) exhibited high interannual variability. The year 2018 was exceptionally wet, particularly in March (266 mm), whereas 2020 and 2022 experienced severe summer droughts, with virtually no rainfall in July–August. In these dry months, cumulative ET_o_ reached its maximum (170.4 mm in July 2020 and 168 mm in July 2022), suggesting critical periods of potential water stress without supplemental irrigation.

Relative humidity (RH) and solar radiation (RS) are shown in Figure 8c. RH displayed a clear inverse seasonal pattern to Rs, with the driest atmosphere recorded in July 2022 (RH = 64.4%), coinciding with peak Rs (27.7 MJ m^−2^ d^−1^ in July 2020). The combination of low RH, high RS, and high ET_o_ highlights increased evaporative demand during the summer, likely intensifying irrigation needs to sustain canopy growth and fruit development.

### 4.3. Fertilization Plan and Treatments

Eight fertilization treatments were applied: two mineral controls (T100, 100% of the conventional dose; T70, 70% of the conventional dose) and six biofertilizer treatments: Mycoshell^®^ (*Glomus* spp. + humic/fulvic acids), Kiplant iNmass^®^ (*Azospirillum brasilense*, *Bacillus megaterium*, and *Saccharomyces cerevisiae*), and Kiplant All-Grip^®^ (*Bacillus megaterium*, *Pseudomonas fluorescens*, and *Pseudomonas putida*). Dosages were defined according to the manufacturers’ agronomic recommendations for fruit trees and adjusted to local soil and climate conditions. Biofertilizers were applied only in combination with 70% of the mineral fertilization dose to evaluate their integrative effects under realistic orchard management conditions. Mycoshell^®^ tablets were placed at the base of each tree at planting, while liquid biofertilizers (iNmass^®^ and All-Grip^®^) were applied annually around the root zone at the recommended doses.

The experiment followed a randomized complete block design with four replications per treatment, each corresponding to one orchard row of 40 trees. Fertilization was carried out using compound fertilizer (15-7-25) and liquid organic matter (Asflow^®^), both supplied by Asfertglobal. Applications were split into two portions during the growing season. Localized mineral fertilization was applied in two phases of the vegetative cycle (T0 and T1) for each treatment. In 2020 and 2021, 50% of each fertilizer product was applied in spring (T0: 7 May 2020; 22 April 2021) and the remaining 50% later in the season (T1: 3 August 2020; 14 June 2021). In 2022, 75% of the total amounts were applied in spring (T0: 6 April 2022) and the remaining 25% in early summer (T1: 14 June 2022). This adjustment accounted for the low soil mobility of phosphorus and potassium and increased nitrogen supply following lower-than-expected productivity in the second year; the revised N application in 2022 improved yields in that year and the following. T100 results from the adjustment of the same fertilizers among the same proportions, with additional nitrogen supplied as ammonium nitrate together with the T1 fertilization. Fertilizer doses were carefully solubilized, weighed, and applied individually to each tree. Only data from 2020–2022 are fully available.

Border rows, potentially influenced by machine traffic and edge effects, were deliberately excluded from treatments to avoid biasing results. Detailed treatment codes, orchard row lines, application rates, and timing are summarized in Table 12, while Table 13 presents the annual fertilization plan, including T0 and T1 percentages, which are available only from 2020 onwards.

### 4.4. Fruit Yield and Quality Assessment

All fruits from 10 trees per treatment were harvested annually from 2019 to 2022. Fruits from each tree were counted and weighed individually, and yield was expressed on an area basis (t ha^−1^). Fruit size distribution was determined, and yield efficiency was calculated as production per unit trunk cross-sectional area (TCSA) [78].

The annual fruit set (%) was calculated as the ratio of fruit set to the number of flowers per corymb from 2019 to 2022. Flower and fruit counts were conducted on two representative corymbs per tree (one from the west quadrant and one from the east quadrant) in ten trees per treatment. Flower counts (at full bloom, BBCH 65) and subsequent fruit counts were performed on the following dates: 11 April and 11 June in 2019, 21 April and 15 June in 2020, 12 April and 2 June in 2021, and 26 April and 27 June in 2022.

Fruit growth was monitored weekly by measuring the maximum (equatorial) diameter of 10 fruits per treatment (one fruit per tree) throughout the growing season using a digital caliper (Calibit, Horticultural Knowledge, Cesena, Italy) to evaluate growth curves and potential treatment effects; measurements on a single fruit per tree were considered sufficient to capture treatment trends. Subsamples of 20–30 fruits per treatment per year, selected to represent average fruit characteristics, were used to assess quality parameters, including fruit weight measured with a digital balance (Baxtran, model BAR, Girona, Spain), diameter and height (Calibit, Horticultural Knowledge, Cesena, Italy), firmness, which was assessed using a bench penetrometer with an 11 mm probe suitable for apples (PENEFEL, Copa–Informatique SA, Yverdon-les-Bains, Switzerland), and total soluble solids (TSS, °Brix) measured with a digital refractometer (HANNA HI96801, HANNA Instruments, Woonsocket, RI, USA).

Dry matter was determined in six replicates per treatment, each consisting of five fruit slices (one slice from each fruit); sampling varied slightly across years due to logistical constraints during the 2020 pandemic and the adoption of a standardized protocol from 2021 onwards. The fresh weight of slices was recorded with an analytical balance (Kern ALJ 160-4AM, max 180 g, precision 0.1 mg; Kern & Sohn GmbH, Ballingen, Germany), and samples were oven-dried at 70 °C until two consecutive measurements indicated constant dry weight.

Color parameters (L*, C*, and Hue) were measured using a Minolta Chroma Meter (CR-300, Konica Minolta, Tokyo, Japan).

*BBI* was calculated to evaluate alternate bearing tendency for each treatment. The *BBI* was computed following the classical formula, adapted from [74]:

(1) $$BBI=\sum_{i=2}^{n}\left[\frac{\text{∣}yi\;+\;yi\;-\;1\text{∣}/(yi\;+\;yi\;-\;1)}{\left(n\;-\;1\right)}\right]$$

yi is the fruit production per tree in year *i* (kg/tree). For each treatment, the yield data were ordered chronologically (2019–2022). The expression calculates the average of the absolute differences between consecutive crops, normalized by the total production of the two crops and by the number of intervals (*n* − 1). BBI values close to 1 indicate high alternation (large variation between years), while values close to 0 indicate regular bearing (low alternation). This method has been applied previously in apple cultivars to assess biennial bearing tendency [74].

### 4.5. Soil and Plant Analysis

Soil samples were collected from the top 0–30 cm layer of the orchard soil on seven dates (5 June 2018, 15 November 2018, 8 May 2019, 29 October 2019, 15 December 2020, 17 February 2022, and 19 December 2022) following the experimental protocol. These dates were chosen to capture both seasonal and interannual dynamics in soil nutrient availability, allowing assessment of treatment effects over time. For each treatment, one composite sample per cycle was obtained by thoroughly mixing several sub-samples collected across the row, ensuring representation of the entire treatment area. Immediately after collection, samples were stored in a thermal box to maintain temperature stability and then transported to an independent accredited laboratory. Upon arrival, samples were air-dried at room temperature (~25 °C) until constant weight was achieved, sieved (<2 mm), and prepared according to ISO 11464:2006 standards [79].

Microbiological analyses of soil were conducted externally by Soilvitae, Lda. at the request of AsfertGlobal. Only two reports, from soil samples collected in June 2019 and July 2020, were made available for this study. Due to the limited dataset and absence of initial and final assessments, the results are reported only as reference information and are not considered sufficient for formal statistical analysis or for drawing conclusions regarding the temporal dynamics of soil microbiology.

Chemical analyses focused on parameters considered most relevant for assessing soil fertility and the potential impact of biofertilizer applications on nutrient dynamics. Analyses included macronutrients (N, %, P, mg/kg; K, mg/kg; Ca, mg/kg; Mg, mg/kg; and S, mg/kg) and micronutrients (Fe, Mn, B, Cu, Zn, Mo, Na, Ni, and Co; mg/kg), as well as pH (H_2_O and CaCl_2_), EC (μS/cm), OM, (%), C/N ratio, and CEC (cmol+/kg). Nutrient concentrations were determined using ICP-OES following Mehlich-3 extraction, while other parameters were measured using standard potentiometric, volumetric, and colorimetric procedures. For consistency in elemental representation, oxide forms reported in the original laboratory bulletins (P_2_O_5_, K_2_O, CaO, and MgO) were converted to elemental forms using the following conversion factors: P_2_O_5_ → P (×0.436), K_2_O → K (×0.830), CaO → Ca (×0.714), and MgO → Mg (×0.603). This approach follows the recommendation that elemental P, K, and Mg should be used instead of oxide forms, as the latter do not occur naturally in soils or plant tissues [80]. Only the most relevant parameters for evaluating the effects of biofertilizer applications are reported here.

Leaf nutrient content was assessed from samples collected on seven dates (16 August 2018, 15 November 2018, 3 June 2019, 31 October 2019, 18 September 2020, 20 July 2021, and 10 August 2022). For each treatment, one composite sample per cycle was obtained from healthy and well-exposed leaves collected from the middle third of current-season shoots. Leaf sampling followed the guidelines of the Integrated Production Manual for Pome Fruits [81], with eight leaves collected per tree from 15 trees per treatment, totaling 120 leaves per unit of sampling. Samples were prepared according to EN 13804:2013 (procedure POF.001). Analyses included macronutrients (N, %, P, %, K, %, Ca, %, Mg, %, and S, %) and micronutrients (Fe, Mn, B, Cu, Zn, Mo, Na, and Al; mg/kg).

### 4.6. Tree Growth and Biometric Measurements

Biometric measurements were conducted on subsets of trees within each treatment to monitor vegetative growth and canopy development from 2018 to 2022. Trunk diameter at 20 cm above the grafting point and annual shoot growth were measured on six trees per treatment, while tree height and canopy volume were recorded on ten trees per treatment. Measurements were performed on specific reference dates to standardize comparisons: 2018—06 June and 15 November; 2019—16 April and 18 September; 2020—08 May and 07 January 2021; 2021—05 April and 07 February 2022; and 2022—11 April and 28 November. Additional measurements were occasionally performed outside these dates to capture growth dynamics and accommodate field and meteorological constraints.

Trunk diameter was measured in three equidistant positions around the trunk using a digital caliper (Calibit, Horticultural Knowledge, Cesena, Italy). The average of the three diameters (*D*1, *D*2, and *D*3) was used to estimate the trunk cross-sectional area (TCSA, cm^2^) according to the formula followed by [21]:

(2) $$\mathrm{TCSA}\;=\;\pi \;\times \;\left(\frac{\;D1\;+\;D2\;+\;D3}{3\;\times \;2}\right)2$$

where *D*1, *D*2, and *D*3 are the trunk diameters measured at 20 cm above the grafting point, π is the mathematical constant (≈3.1416), and the denominator reflects average radius.

Tree height (TH) and canopy volume (CV) were estimated using standard field measurements. Total tree height and height to the base of the canopy were measured with a flexible measuring tape, while canopy width was measured at three vertical positions: base, mid-height, and approximately 50 cm below the top of the canopy. Canopy volume (V, m^3^) was calculated using the standard formula provided by [21]:

(3) $$\mathrm{CV}\;=\;\left(\frac{\;L_{\mathrm{base}}\;+\;L_{\mathrm{middle}}\;+\;L_{\mathrm{top}}}{3\;\times \;2}\right)\text{÷}100\;\times (H_{\mathrm{total}}-H_{\mathrm{base}})$$

where L_base,_ L_middle_, and L_top_ are canopy widths (cm) and H_total_ and H_base_ are measured in meters. Canopy volume measurements were initiated in 2020, as no data were collected prior to this year.

### 4.7. Statistical Analysis

Data for soil chemical properties and leaf nutrient contents (2018–2022) were analyzed descriptively to identify temporal trends and treatment effects, focusing on consistency and patterns rather than formal statistical comparisons. Because each fertilization treatment was applied to a single orchard row, individual trees were considered sub-samples rather than independent replicates; therefore, treatment-level comparisons for soil and leaf data are exploratory, and ANOVA was not applied. PCA was conducted separately for soil and leaf parameters to explore multivariate relationships among standardized variables. Components with eigenvalues >1 were retained, and Promax rotation was applied. Loadings ≥0.4 were considered relevant and ≥0.7 strong contributors. Sampling adequacy and sphericity were verified using the KMO test and Bartlett’s test of sphericity, respectively, to ensure suitability of the data for PCA.

To further evaluate whether treatments differed in their multivariate soil profiles, one-way ANOVA was applied to the scores of the first four principal components. Tree biometric parameters—including tree height (2018–2022), canopy volume (2020–2022), TCSA (2018–2022), and shoot growth (2018–2022)—as well as fruit set (2019–2022), fruit yield and mean fruits per tree (2019–2022), and fruit physical and quality parameters (diameter, weight, firmness, soluble solids, dry matter, and hue; 2019–2022) were analyzed using one-way ANOVA. Homogeneity of variance was assessed with Levene’s test. When variances were unequal, Games–Howell post hoc tests were applied [82].

Fruit size distribution data violated assumptions of normality and homoscedasticity and were therefore analyzed using the Kruskal–Wallis test, followed by Dunn’s post hoc pairwise comparisons with Bonferroni correction to control for multiple testing.

Additionally, relationships among tree and fruit production parameters (number of fruits per tree, average fruit weight, TCSA, and yield per TCSA) were assessed using Pearson correlation analysis. Only statistically significant correlations (*p* < 0.05) were considered relevant.

All analyses were performed using JASP version 0.19.1 (University of Amsterdam, Amsterdam, The Netherlands).

## 5. Conclusions

Biofertilizer applications exert measurable effects on soil chemical fertility, foliar nutrient composition, and vegetative growth of mature apple trees under Mediterranean conditions. Over five years, biofertilizer treatments enhanced soil K, Mg, Zn, and Cu, promoted canopy expansion, trunk thickening, and shoot elongation, and maintained leaf nutrient levels within optimal ranges, while conventional mineral fertilization only (T100 and T70) better sustained leaf nitrogen. Fruit yield and quality responses were strongly influenced by inter-annual climatic variability, with climate dominating production outcomes, while fertilization treatments functioned as modulators rather than primary drivers. Mineral treatments produced larger fruits, whereas biofertilizers promoted a more balanced distribution of commercially valuable medium-sized fruits. Within the orchard’s permanent living mulch system, microbial inoculant-based treatments consistently improved vegetative growth, suggesting synergistic effects between soil cover, nutrient cycling, and tree vigor. Enhanced vegetative growth supports the establishment of less branched, higher-yielding trees and may mitigate climate-related stresses, rather than directly increasing total yield.

Overall, biofertilizer-based nutrient programs can partially modulate the effects of mineral fertilization without compromising fruit quality, supporting soil health, resilience to climatic fluctuations, and sustainable orchard management, in line with the European Union’s Farm to Fork strategy. A key limitation of this study is the lack of independent replication at the treatment level, as each fertilization treatment was implemented in a single orchard row; individual trees therefore serve as sub-samples rather than fully independent replicates. Future research should focus on multi-site trials, longer-term assessments, water use efficiency, and economic analyses to quantify cost savings and optimize integrated nutrient–water management strategies in precision fruit production.

## Figures and Tables

**Table 1:** Fruit set percentage (“Vingamento”) in apple trees over four years (2019–2022) under different fertilization treatments. Values are means ± standard error of 20 clusters per treatment. Superscript letters indicate significant differences among treatments within each year (Games–Howell post hoc, *p* < 0.05).

Treatment	2019	2020	2021	2022
T100	21.51 ± 3.50 ^a^	13.67 ± 4.04 ^a^	8.51 ± 2.60 ^a^	15.93 ± 2.86 ^a^
Myc2	13.63 ± 3.52 ^a^	18.33 ± 6.14 ^a^	4.34 ± 1.73 ^a^	7.72 ± 2.61 ^a^
Myc4	30.47 ± 4.85 ^a^	12.51 ± 4.08 ^a^	7.68 ± 2.86 ^a^	9.80 ± 2.79 ^a^
iNM6	25.49 ± 3.07 ^a^	7.68 ± 2.30 ^a^	7.03 ± 2.95 ^a^	14.51 ± 2.80 ^a^
iNM12	16.96 ± 3.59 ^a^	6.00 ± 3.51 ^a^	6.34 ± 3.24 ^a^	10.84 ± 2.03 ^a^
Allg12	25.57 ± 4.03 ^a^	9.01 ± 3.29 ^a^	7.85 ± 2.00 ^a^	12.51 ± 2.56 ^a^
Allg6	22.93 ± 3.64 ^a^	10.02 ± 4.25 ^a^	8.55 ± 3.12 ^a^	9.18 ± 2.56 ^a^
T70	28.95 ± 5.43 ^a^	11.94 ± 3.36 ^a^	13.00 ± 4.10 ^a^	15.47 ± 3.92 ^a^

**Table 2:** Fruit production under different fertilization treatments over 2019–2022. Values are means ± standard error. Columns show fruits per tree, average fruit weight (g), and fruit production per tree (kg) and per hectare (t/ha). Superscript letters indicate significant differences among treatments within each year (Games–Howell post hoc, *p* < 0.05).

Treatment	Year	Number of Fruits per Tree	Avg. Fruit Weight (g)	Fruit Production (kg/tree)
T100	2019	42.0 ± 3.65 ^a^^b^	145 ± 5.30 ^b^	6.07 ± 0.53 ^a^^b^
	2020	44.8 ± 4.88 ^c^	121 ± 5.42 ^a^	6.27 ± 0.60 ^c^
	2021	57.8 ± 5.84 ^a^	131 ± 5.43 ^a^^b^	8.46 ± 0.68 ^b^^c^
	2022	66.3 ± 4.02 ^a^^b^	137 ± 3.04 ^a^^b^	8.31 ± 0.49 ^a^^b^
Myc2	2019	59.8 ± 4.72 ^c^	141 ± 3.34 ^a^	7.15 ± 0.54 ^b^
	2020	35.6 ± 2.51 ^b^^c^	132 ± 3.65 ^a^^b^	4.79 ± 0.30 ^b^
	2021	48.8 ± 5.63 ^a^	131 ± 5.99 ^a^^b^	7.47 ± 0.87 ^a^^b^
	2022	62.8 ± 5.94 ^a^^b^	130 ± 3.86 ^a^^b^	8.26 ± 0.80 ^a^^b^
Myc4	2019	55.2 ± 5.39 ^b^^c^	141 ± 5.99 ^a^^b^	7.04 ± 0.54 ^b^
	2020	29.1 ± 4.17 ^a^^b^	135 ± 2.99 ^a^	3.90 ± 0.47 ^a^
	2021	57.7 ± 6.45 ^a^	138 ± 4.67 ^a^	9.07 ± 1.08 ^c^
	2022	84.4 ± 6.24 ^b^	144 ± 4.16 ^b^	10.43 ± 0.79 ^c^
iNM6	2019	51.8 ± 3.67 ^b^^c^	139 ± 2.68 ^a^	6.96 ± 0.30 ^b^
	2020	31.4 ± 1.96 ^a^^b^	156 ± 3.59 ^b^	4.58 ± 0.42 ^b^
	2021	52.3 ± 5.48 ^a^	150 ± 5.97 ^a^^b^	8.33 ± 0.73 ^b^^c^
	2022	69.8 ± 5.22 ^a^^b^	144 ± 6.45 ^a^^b^	9.00 ± 0.54 ^c^
iNM12	2019	37.2 ± 2.64 ^a^	151 ± 6.83 ^a^^b^	5.19 ± 0.33 ^a^
	2020	30.1 ± 1.65 ^a^^b^	154 ± 8.67 ^a^^b^	4.18 ± 0.27 ^b^
	2021	53.6 ± 6.82 ^a^	157 ± 6.47 ^a^^b^	7.48 ± 0.79 ^a^^b^
	2022	54.0 ± 5.62 ^a^^b^	164 ± 7.26 ^a^^b^	6.76 ± 0.67 ^a^
Allg6	2019	37.4 ± 2.88 ^a^	144 ± 7.89 ^a^^b^	4.91 ± 0.37 ^a^
	2020	19.8 ± 2.53 ^a^	158 ± 3.53 ^a^^b^	3.12 ± 0.36 ^a^
	2021	42.4 ± 3.47 ^a^	146 ± 4.04 ^a^^b^	6.63 ± 0.45 ^a^
	2022	51.6 ± 3.76 ^a^	133 ± 5.92 ^a^	7.25 ± 0.48 ^a^
Allg12	2019	59.5 ± 4.19 ^c^	126 ± 4.85 ^a^^b^	7.69 ± 0.45 ^b^
	2020	20.3 ± 2.83 ^a^	131 ± 8.04 ^a^^b^	2.96 ± 0.36 ^a^
	2021	51.5 ± 5.07 ^a^	124 ± 3.86 ^b^	7.46 ± 0.67 ^a^^b^
	2022	65.5 ± 5.69 ^a^^b^	131 ± 5.42 ^a^^b^	8.52 ± 0.53 ^a^^b^
T70	2019	51.7 ± 3.65 ^b^^c^	126 ± 6.65 ^a^^b^	6.62 ± 0.39 ^a^^b^
	2020	39.6 ± 4.68 ^b^	141 ± 4.77 ^a^^b^	5.68 ± 0.65 ^b^^c^
	2021	65.8 ± 6.69 ^a^	133 ± 3.97 ^a^^b^	8.39 ± 0.59 ^b^^c^
	2022	58.9 ± 6.38 ^a^^b^	130 ± 0.92 ^a^^b^	7.74 ± 0.92 ^a^^b^

**Table 3:** Pearson correlation coefficients between tree and fruit parameters. Only statistically significant correlations (*p* < 0.05) are shown.

Variable 1	Variable 2	Pearson’s r	*p*-Value
Fruits per tree	Avg. fruit weight	−0.44	<0.001
TCSA	Kg per TCSA	−0.73	<0.001

**Table 4:** Fruit quality parameters under different fertilization treatments over 2019–2022. Values are means ± standard error. Columns show diameter (mm), weight (g), firmness (kg/cm^2^), brix (°), dry matter (%) and hue (°). Superscript letters indicate significant differences among treatments within each year (Games–Howell post hoc, *p* < 0.05).

Treatment	Year	Diameter (mm)	Weight (g)	Firmness (kg/cm^2^)	Brix (°)	Dry Matter (%)	Hue (°)
T100	2019	70.91 ± 0.71 ^a^	148.74 ± 4.44 ^a^	8.08 ± 0.16 ^a^	12.93 ± 0.21 ^a^	16.50 ± 0.25 ^a^	40.39 ± 2.37 ^b^
	2020	71.42 ± 0.66 ^a^	151.46 ± 4.13 ^a^	7.76 ± 0.20 ^a^	14.69 ± 0.29 ^a^	17.48 ± 0.23 ^a^	30.78 ± 0.91 ^a^
	2021	68.46 ± 0.38 ^a^^b^^c^	144.95 ± 2.15 ^a^^b^	8.51 ± 0.30 ^a^^b^	14.42 ± 0.24 ^a^^b^	17.58 ± 0.31 ^a^^b^	30.78 ± 0.91 ^a^
	2022	67.55 ± 0.21 ^a^^b^	129.44 ± 1.41 ^a^^b^	8.43 ± 0.20 ^a^^b^	18.17 ± 0.26 ^a^	18.67 ± 0.24 ^a^	30.78 ± 0.91 ^c^
Myc2	2019	69.51 ± 0.69 ^a^	136.75 ± 3.45 ^a^	8.29 ± 0.14 ^a^	13.74 ± 0.27 ^a^^b^	17.45 ± 0.30 ^a^	34.09 ± 1.23 ^c^
	2020	73.53 ± 0.73 ^a^	163.58 ± 5.34 ^a^	7.70 ± 0.16 ^a^	15.22 ± 0.22 ^a^	18.00 ± 1.15 ^a^	34.09 ± 1.23 ^a^
	2021	69.77 ± 0.52^c^	155.53 ± 3.73 ^b^	8.22 ± 0.31 ^a^^b^	14.56 ± 0.26 ^b^	17.47 ± 0.15 ^b^	34.09 ± 1.23 ^a^
	2022	66.99 ± 0.33 ^a^	129.10 ± 3.11 ^a^	8.82 ± 0.22 ^b^	13.90 ± 0.25 ^a^	17.38 ± 0.17 ^a^	34.09 ± 1.23 ^a^^b^
Myc4	2019	70.60 ± 0.56 ^a^	143.96 ± 3.32 ^a^	8.47 ± 0.30 ^a^	14.19 ± 0.27 ^b^	17.11 ± 0.27 ^a^	31.93 ± 1.39 ^b^
	2020	72.06 ± 0.65 ^a^	161.80 ± 4.42 ^a^	7.84 ± 0.24 ^a^	14.92 ± 0.36 ^a^	17.50 ± 0.14 ^a^	31.93 ± 1.39 ^a^
	2021	69.32 ± 0.39 ^b^^c^	148.56 ± 3.36 ^a^^b^	9.29 ± 0.20 ^b^	13.21 ± 0.25 ^a^	16.82 ± 0.20 ^c^	31.93 ± 1.39 ^a^
	2022	67.86 ± 0.23 ^a^^b^	136.97 ± 1.50 ^b^	7.61 ± 0.15 ^a^	14.13 ± 0.20 ^a^	18.22 ± 0.41 ^a^	31.93 ± 1.39 ^b^
iNM6	2019	70.84 ± 0.59 ^a^	145.87 ± 3.32 ^a^	8.30 ± 0.18 ^a^	13.84 ± 0.21 ^a^^b^	17.40 ± 0.13 ^a^	36.53 ± 2.27 ^a^^b^
	2020	72.86 ± 0.70 ^a^	162.82 ± 4.28 ^a^	7.89 ± 0.22 ^a^	15.18 ± 0.25 ^a^	17.32 ± 0.17 ^a^	36.53 ± 2.27 ^a^
	2021	68.29 ± 0.47 ^a^^b^^c^	142.81 ± 2.83 ^a^	8.32 ± 0.25 ^a^^b^	13.75 ± 0.26 ^a^^b^	16.98 ± 0.08 ^b^	36.53 ± 2.27 ^a^
	2022	66.99 ± 0.22 ^a^	128.39 ± 1.58 ^a^	8.27 ± 0.19 ^a^^b^	14.22 ± 0.20 ^a^	18.20 ± 0.51 ^a^	36.53 ± 2.27 ^a^^b^
iNM12	2019	71.60 ± 0.53 ^a^	151.55 ± 3.61 ^a^	8.80 ± 0.17 ^a^^b^	14.49 ± 0.31 ^b^	17.46 ± 0.14 ^a^	32.01 ± 0.85 ^a^^b^
	2020	72.37 ± 0.52 ^a^	159.01 ± 3.92 ^a^	7.74 ± 0.26 ^a^	15.20 ± 0.37 ^a^	17.72 ± 0.34 ^a^	32.01 ± 0.85 ^a^
	2021	67.42 ± 0.30 ^a^	138.83 ± 2.24 ^a^	8.51 ± 0.23 ^a^^b^	14.44 ± 0.25 ^b^	18.55 ± 0.31 ^c^	32.01 ± 0.85 ^a^
	2022	67.95 ± 0.21 ^a^^b^	132.22 ± 1.19 ^a^^b^	8.19 ± 0.21 ^a^^b^	14.61 ± 0.28 ^a^	18.09 ± 0.32 ^a^	32.01 ± 0.85 ^b^
Allg6	2019	70.44 ± 0.57 ^a^	141.98 ± 3.57 ^a^	8.67 ± 0.20 ^a^^b^	14.80 ± 0.24 ^b^	17.41 ± 0.14 ^a^	32.59 ± 1.15 ^b^
	2020	72.80 ± 0.66 ^a^	157.69 ± 4.12 ^a^	7.83 ± 0.22 ^a^	15.46 ± 0.26 ^a^	17.62 ± 0.40 ^a^	32.59 ± 1.15 ^a^
	2021	67.63 ± 0.35 ^a^^b^	139.43 ± 2.36 ^a^	8.39 ± 0.28 ^a^^b^	14.00 ± 0.26 ^a^^b^	18.01 ± 0.15 ^b^^c^	32.59 ± 1.15 ^a^
	2022	67.90 ± 0.19 ^a^^b^	134.40 ± 1.79 ^a^^b^	8.11 ± 0.21 ^a^^b^	14.63 ± 0.28 ^a^	17.86 ± 0.18 ^a^	32.59 ± 1.15 ^a^^b^
Allg12	2019	70.12 ± 0.51 ^a^	139.01 ± 2.51 ^a^	8.51 ± 0.16 ^a^	13.87 ± 0.28 ^a^^b^	17.31 ± 0.19 ^a^	38.45 ± 2.25 ^a^
	2020	72.83 ± 0.69 ^a^	159.59 ± 3.85 ^a^	7.38 ± 0.23 ^a^	15.92 ± 0.26 ^a^	17.67 ± 0.21 ^a^	38.45 ± 2.25 ^a^
	2021	68.52 ± 0.53 ^a^^b^^c^	146.50 ± 2.88 ^a^^b^	8.85 ± 0.24 ^a^^b^	13.86 ± 0.20 ^a^^b^	18.17 ± 0.26 ^c^	38.45 ± 2.25 ^a^
	2022	67.22 ± 0.20 ^a^^b^	127.18 ± 1.50 ^a^	8.26 ± 0.22 ^a^^b^	14.61 ± 0.28 ^a^	18.16 ± 0.26 ^a^	38.45 ± 2.25 ^b^^c^
T70	2019	70.36 ± 0.71 ^a^	143.49 ± 3.74 ^a^	9.36 ± 0.18 ^b^	14.07 ± 0.29 ^a^^b^	17.17 ± 0.15 ^a^	30.42 ± 0.91 ^b^
	2020	72.03 ± 0.38 ^a^	154.47 ± 2.75 ^a^	7.46 ± 0.23 ^a^	15.07 ± 0.34 ^a^	16.72 ± 0.14 ^a^	30.42 ± 0.91 ^a^
	2021	68.40 ± 0.35 ^a^^b^^c^	143.49 ± 2.70 ^a^^b^	8.11 ± 0.19 ^a^	13.31 ± 0.28 ^a^	18.35 ± 0.38 ^c^	30.42 ± 0.91 ^a^
	2022	68.12 ± 0.22 ^b^	134.55 ± 1.52 ^a^^b^	8.05 ± 0.19 ^a^^b^	14.35 ± 0.30 ^a^	17.32 ± 0.17 ^a^	30.42 ± 0.91 ^a^

**Table 5:** Rotated component loadings from PCA of standardized soil parameters (RC1–RC4). Only loadings ≥0.4 are shown; strong loadings (≥0.7) are in bold. Promax rotation applied.

Comp.	Eigenvalue	% Var (Rot)	Cumulative %	Key Vars (>0.6)
RC1	2.991	18.7%	18.7%	S (0.827), EC (0.779), B (0.711), Fe (0.701), Mg (0.687)
RC2	2.777	17.4%	36.0%	pH (H_2_O 0.938, CaCl_2_ 0.935), Ca (0.466)
RC3	2.598	16.2%	52.3%	Zn (0.873), K (0.642), OM (−0.456), CEC (−0.596), Cu (0.536)
RC4	2.182	13.6%	65.9%	N (0.884), C/N (−0.831), P (0.677)

Comp. = component; Eigenvalue = variance explained; % Var (Rot) = percentage of variance explained after rotation; Cum. % = cumulative variance; Key Vars (>0.6) = major contributing variables. Parameter units are grouped as follows: N, K, Mg, Ca, and OM in %; P, S, Fe, B, and Zn in mg/kg; CEC in cmol(+)/kg; C/N is unitless; pH measured in water (H_2_O) and in 0.01 M CaCl_2_; EC in µS/cm.

**Table 6:** Mean ± standard deviation of the scores for principal components PC1–PC4 by treatment. Significance was evaluated using one-way ANOVA; ns indicates no significant differences, and * indicates significant differences among treatments (*p* < 0.05).

Treatment	PC1	PC2	PC3	PC4	Significance (*p*)
T100	0.077 ± 1.153	0.126 ± 1.112	0.378 ± 1.184	0.571 ± 1.233	ns/ns/*/*
Myc2	0.058 ± 1.112	0.032 ± 1.246	−0.235 ± 0.884	−0.114 ± 1.044	ns/ns/ns/ns
Myc4	−0.061 ± 1.133	−0.051 ± 1.092	0.068 ± 0.956	−0.092 ± 1.017	ns/ns/ns/ns
iNM6	0.045 ± 1.140	0.056 ± 1.264	0.037 ± 1.050	−0.081 ± 0.943	ns/ns/ns/ns
iNM12	−0.010 ± 1.040	−0.194 ± 0.856	0.123 ± 1.168	0.069 ± 1.156	ns/ns/ns/ns
Allg6	0.026 ± 0.906	−0.024 ± 0.970	−0.173 ± 0.986	−0.158 ± 0.985	ns/ns/ns/ns
Allg12	−0.031 ± 1.130	0.039 ± 0.959	0.040 ± 1.027	−0.112 ± 1.124	ns/ns/ns/ns
T70	−0.105 ± 0.897	0.017 ± 0.960	−0.236 ± 1.104	−0.162 ± 0.978	ns/ns/ns/ns

**Table 7:** Cumulative percentage change (%) in soil parameters between 2018 and 2022 for each treatment.

∆_Period	T	pH (H_2_O)	pH (CaCl_2_)	EC	OM	N	C/N	P	K
22−18	T100	25% ▲	19% ▲	−13% *▼*	−2% →	33% ▲	−23% *▼*	99% ▲	95% ▲
22−18	Myc2	25% ▲	20% ▲	27% ▲	−9% *▼*	0% →	−9% *▼*	101% ▲	78% ▲
22−18	Myc4	25% ▲	19% ▲	20% ▲	−17% *▼*	−11% *▼*	−11% *▼*	58% ▲	65% ▲
22−18	iNM6	20% ▲	20% ▲	80% ▲	−28% *▼*	−11% *▼*	−18% *▼*	0% →	39% ▲
22−18	iNM12	20% ▲	18% ▲	56% ▲	−11% *▼*	11% ▲	−18% *▼*	1445% ▲	49% ▲
22−18	Allg6	22% ▲	17% ▲	44% ▲	−19% *▼*	−21% *▼*	7% ▲	21% ▲	18% ▲
22−18	Allg12	19% ▲	14% ▲	2% →	−25% *▼*	−29% *▼*	10% ▲	27% ▲	34% ▲
22−18	T70	24% ▲	19% ▲	53% ▲	−18% *▼*	−36% *▼*	21% ▲	1% →	18% ▲
∆_Period	T	Ca	Mg	S	Fe	B	Cu	Zn	CEC
22−18	T100	42% ▲	67% ▲	34% ▲	−11% *▼*	44% ▲	45% ▲	97% ▲	−12%*▼*
22−18	Myc2	43% ▲	67% ▲	101% ▲	−2% →	46% ▲	39% ▲	86% ▲	−19% *▼*
22−18	Myc4	31% ▲	60% ▲	−48% *▼*	−13% *▼*	7% ▲	1% →	55% ▲	−19% *▼*
22−18	iNM6	23% ▲	20% ▲	261% ▲	2% →	23% ▲	1% →	28% ▲	−21% *▼*
22−18	iNM12	28% ▲	23% ▲	198% ▲	6% ▲	47% ▲	−4% →	413% ▲	−7% *▼*
22−18	Allg6	28% ▲	56% ▲	−14% *▼*	−29% *▼*	24% ▲	20% ▲	55% ▲	−19% *▼*
22−18	Allg12	15% ▲	26% ▲	−23% *▼*	−21% *▼*	20% ▲	18% ▲	42% ▲	−19% *▼*
22−18	T70	24% ▲	37% ▲	−29% *▼*	−29% *▼*	26% ▲	−5% →	6% ▲	−2% →

Symbols indicate the direction and relative magnitude of change: ▲ = increase ≥ +21% (dark green); ▲ = increase +6% to +20% (light green); → = stability (−5% to +5%); ▼ = decrease −6% to −20% (orange); ▼ = decrease ≤ −21% (red). T = applied fertilization or biofertilizer treatment. Parameter units: pH and C/N (unitless); EC (µS/cm); OM and N (%); P, K, Ca, Mg, S, Fe, Zn, B, Cu (mg/kg); CEC (cmol(+)/kg).

**Table 8:** Rotated component loadings from PCA of standardized leaf parameters. Only loadings ≥0.4 are shown; strong loadings (≥0.7) are highlighted in bold. Eigenvalues, percentage of variance explained, cumulative variance, and key variables with loadings greater than |0.6| are shown for each component (RC1–RC3). Promax rotation was applied.

Comp.	Eigenvalue	% Var (Rot)	Cum. %	Key Vars (>0.6)
RC1	5.014	35.8%	35.8%	Zn (0.916), Fe (−0.885), Al (−0.797), P (0.775), N (0.745), B (0.708)
RC2	2.608	18.6%	54.4%	Mn (0.646), Ca (−0.525), N (0.582)
RC3	2.489	17.8%	72.2%	Cu (0.865), K (0.773), S (−0.671), Na (−0.932), Mg (0.905)

Comp. = component; Eigenvalue = variance explained; % Var (Rot) = variance % after rotation; Cum. % = cumulative variance %; Key Vars (>0.6) = major contributing nutrients. Nutrients: N (%), P (%), K (%), Ca (%), Mg (%), S (%), Fe (mg/kg), Mn (mg/kg), B (mg/kg), Cu (mg/kg), Zn (mg/kg), Na (mg/kg), and Al (mg/kg).

**Table 9:** Mean ± standard deviation of the scores for the first three principal components (PC1–PC3) for each treatment. Significance was assessed by one-way ANOVA; ns indicates no significant differences among treatments (*p* > 0.05).

Treatment	PC1	PC2	PC3	Significance (*p*)
T100	0.077 ± 1.153	0.126 ± 1.112	0.378 ± 1.184	ns
Myc2	0.058 ± 1.112	0.032 ± 1.246	−0.235 ± 0.884	ns
Myc4	−0.061 ± 1.133	−0.051 ± 1.092	0.068 ± 0.956	ns
iNM6	0.045 ± 1.140	0.056 ± 1.264	0.037 ± 1.050	ns
iNM12	−0.010 ± 1.040	−0.194 ± 0.856	0.123 ± 1.168	ns
Allg6	0.026 ± 0.906	−0.024 ± 0.970	−0.173 ± 0.986	ns
Allg12	−0.031 ± 1.130	0.039 ± 0.959	0.040 ± 1.027	ns
T70	−0.105 ± 0.897	0.017 ± 0.960	−0.236 ± 1.104	ns

**Table 10:** Cumulative percentage change (%) in leaf parameters between 2018 and 2022 for each treatment.

∆_Period	T	N	P	K	Ca	Mg	S	Fe
22−18	T100	−16% ▼	−50% ▼	27% ▲	29% ▲	0% →	−67% ▼	119% ▲
22−18	Myc2	−46% ▼	−50% ▼	54% ▲	100% ▲	0% →	−50% ▼	180% ▲
22−18	Myc4	−48% ▼	−50% ▼	31% ▲	89% ▲	33% ▲	−50% ▼	210% ▲
22−18	iNM6	−53% ▼	−50% ▼	27% ▲	73% ▲	0% →	−50% ▼	182% ▲
22−18	iNM12	−54% ▼	−50% ▼	13% ▲	25% ▲	0% →	−50% ▼	174% ▲
22−18	Allg6	−53% ▼	0% →	91% ▲	88% ▲	0% →	−50% ▼	188% ▲
22−18	Allg12	−57% ▼	−50% ▼	7% ▲	45% ▲	−25% ▼	−50% ▼	186% ▲
22−18	T70	−30% ▼	0% →	47% ▲	29% ▲	−25% ▼	−67% ▼	145% ▲
∆_Period	T	Mn	B	Cu	Zn	Mo	Na	Al
22−18	T100	−79% ▼	−23% ▼	−13% ▼	−67% ▼	−67% ▼	500% ▲	306% ▲
22−18	Myc2	−89% ▼	−19% ▼	76% ▲	−69% ▼	−80% ▼	101% ▲	470% ▲
22−18	Myc4	−82% ▼	−13% ▼	5% →	−68% ▼	−80% ▼	−32% ▼	426% ▲
22−18	iNM6	−84% ▼	−18% ▼	9% ▲	−73% ▼	−50% ▼	321% ▲	398% ▲
22−18	iNM12	−82% ▼	−31% ▼	5% →	−72% ▼	−75% ▼	450% ▲	323% ▲
22−18	Allg6	−83% ▼	−7% ▼	12% ▲	−71% ▼	−33% ▼	400% ▲	330% ▲
22−18	Allg12	−86% ▼	−30% ▼	12% ▲	−73% ▼	−50% ▼	400% ▲	350% ▲
22−18	T70	−91% ▼	−23% ▼	−20% ▼	−77% ▼	33% ▲	450% ▲	205% ▲

Symbols indicate descriptive trends: ▲ = increase ≥ +21% (dark green);▲ = increase +6% to +20% (light green); → = stability (−5% to +5%); ▼ = decrease −6% to −20% (orange); ▼ = decrease ≤ −21% (red). Nutrient units: N, P, K, Ca, Mg, and S = %; Fe, Mn, B, Cu, Zn, Mo, Na, Al, and Cl = mg/kg.

**Table 11:** Height (m), canopy volume (m^3^), TCSA (cm^2^), shoot growth (m) and cumulative shoot growth (m) of apple trees under different fertilization treatments over the study period (2018–2022). Values are presented as mean ± standard error. Canopy volume was measured only in 2020, 2021, and 2022, hence “nd” indicates data not measured in other years. Superscript letters indicate significant differences among treatments within the same year (Games–Howell post hoc, *p* < 0.05).

Treatment	Year	Height (m)	CV (m^3^)	TCSA (cm^2^)	Shoot Growth (m)	Cumulative Shoot Growth (m)
T100	2018	1.95 ± 0.07 ^a^	nd	2.09 ± 0.18 ^a^	2.65 ± 0.50 ^a^^b^	–
	2019	2.14 ± 0.03 ^a^	nd	3.29 ± 0.13 ^a^	6.15 ± 0.26 ^a^^b^	–
	2020	2.33 ± 0.04 ^a^	1.78 ± 0.07 ^a^^b^	4.39 ± 0.51 ^a^	2.73 ± 0.48 ^a^	–
	2021	1.59 ± 0.25 ^a^	1.19 ± 0.06 ^a^	7.65 ± 0.34 ^a^	13.83 ± 0.93 ^a^	–
	2022	2.74 ± 0.05 ^a^	1.33 ± 0.08 ^a^	9.15 ± 0.29 ^a^	19.32 ± 2.15 ^a^	44.68 ± 2.45
Myc2	2018	2.16 ± 0.03 ^b^	nd	3.09 ± 0.25 ^c^	3.60 ± 0.93 ^a^^b^	–
	2019	2.23 ± 0.02 ^a^^b^	nd	4.30 ± 0.08 ^c^	7.11 ± 0.37 ^b^	–
	2020	2.43 ± 0.03 ^a^	2.09 ± 0.09 ^b^^c^	4.84 ± 0.59 ^a^	2.48 ± 0.51 ^a^	–
	2021	1.59 ± 0.24 ^a^	1.18 ± 0.06 ^a^	8.63 ± 0.34 ^b^	15.36 ± 1.91 ^a^	–
	2022	2.72 ± 0.02 ^a^	1.29 ± 0.07 ^a^	10.11 ± 0.33 ^a^	23.60 ± 1.00 ^a^	52.15 ± 2.16
Myc4	2018	2.10 ± 0.04 ^a^^b^	nd	3.00 ± 0.23 ^b^^c^	3.99 ± 0.47 ^b^	–
	2019	2.22 ± 0.02 ^a^^b^	nd	4.36 ± 0.12 ^c^	6.84 ± 0.55 ^b^	–
	2020	2.45 ± 0.05 ^a^	2.21 ± 0.12 ^c^	5.40 ± 0.75 ^a^	6.76 ± 1.51 ^b^	–
	2021	1.61 ± 0.25 ^a^	1.30 ± 0.07 ^a^	9.97 ± 0.49 ^b^	20.77 ± 2.05 ^a^	–
	2022	2.86 ± 0.06 ^a^	1.46 ± 0.11 ^a^	11.91 ± 0.52 ^b^	30.43 ± 3.55 ^a^	68.79 ± 4.06
iNM6	2018	2.06 ± 0.04 ^a^^b^	nd	2.36 ± 0.15 ^a^^b^^c^	1.99 ± 0.27 ^a^^b^	–
	2019	2.16 ± 0.03 ^a^	nd	3.44 ± 0.09 ^a^^b^	6.28 ± 0.31 ^b^	–
	2020	2.37 ± 0.05 ^a^	1.86 ± 0.07 ^a^^b^	4.87 ± 0.62 ^a^	4.92 ± 0.67 ^b^	–
	2021	1.58 ± 0.25 ^a^	1.23 ± 0.06 ^a^	8.66 ± 0.37 ^a^^b^	18.49 ± 1.19 ^a^	–
	2022	2.80 ± 0.04 ^a^	1.49 ± 0.08 ^a^	10.28 ± 0.33 ^a^	28.57 ± 4.03 ^a^	60.25 ± 4.28
iNM12	2018	2.01 ± 0.04 ^a^^b^	nd	2.26 ± 0.17 ^a^^b^	2.22 ± 0.35 ^a^^b^	–
	2019	2.17 ± 0.02 ^a^^b^	nd	3.54 ± 0.12 ^b^	6.22 ± 0.75 ^b^	–
	2020	2.35 ± 0.04 ^a^	1.80 ± 0.07 ^a^^b^	4.86 ± 0.66 ^a^	5.09 ± 0.70 ^b^	–
	2021	1.57 ± 0.25 ^a^	1.26 ± 0.05 ^a^	8.01 ± 0.36 ^a^	17.38 ± 2.26 ^a^	–
	2022	2.75 ± 0.05 ^a^	1.41 ± 0.08 ^a^	9.39 ± 0.38 ^a^	23.03 ± 2.14 ^a^	53.94 ± 2.45
Allg6	2018	2.13 ± 0.02 ^b^	nd	2.13 ± 0.13 ^a^	1.61 ± 0.52 ^a^	–
	2019	2.20 ± 0.02 ^a^^b^	nd	3.26 ± 0.11 ^a^	4.32 ± 0.26 ^a^^b^	–
	2020	2.32 ± 0.03 ^a^	1.70 ± 0.07 ^a^	4.62 ± 0.57 ^a^	4.13 ± 0.85 ^b^	–
	2021	1.55 ± 0.24 ^a^	1.17 ± 0.08 ^a^	8.27 ± 0.32 ^a^	15.06 ± 2.39 ^a^	–
	2022	2.67 ± 0.05 ^a^	1.15 ± 0.08 ^a^	9.99 ± 0.37 ^a^	21.05 ± 1.71 ^a^	46.17 ± 2.98
Allg12	2018	2.09 ± 0.04 ^a^^b^	nd	2.54 ± 0.16 ^a^^b^^c^	2.74 ± 0.32 ^a^^b^	–
	2019	2.23 ± 0.02 ^a^^b^	nd	3.61 ± 0.08 ^a^^b^	6.60 ± 0.48 ^b^	–
	2020	2.40 ± 0.03 ^a^	1.80 ± 0.07 ^b^^c^	4.84 ± 0.58 ^a^	2.86 ± 0.70 ^a^	–
	2021	1.60 ± 0.25 ^a^	1.28 ± 0.05 ^a^	8.35 ± 0.25 ^a^	17.38 ± 2.22 ^a^	–
	2022	2.79 ± 0.05 ^a^	1.43 ± 0.08 ^a^	9.81 ± 0.23 ^a^	23.52 ± 0.97 ^a^	53.10 ± 2.59
T70	2018	2.13 ± 0.02 ^b^	nd	2.47 ± 0.16 ^a^^b^^c^	2.26 ± 0.22 ^a^^b^	–
	2019	2.27 ± 0.02 ^b^	nd	3.78 ± 0.12 ^b^	6.60 ± 0.54 ^b^	–
	2020	2.43 ± 0.03 ^a^	2.13 ± 0.08 ^b^^c^	4.63 ± 0.56 ^a^	3.04 ± 0.86 ^a^	–
	2021	1.64 ± 0.23 ^a^	1.23 ± 0.05 ^a^	7.93 ± 0.33 ^a^	14.62 ± 1.56 ^a^	–
	2022	2.74 ± 0.03 ^a^	1.31 ± 0.08 ^a^	9.19 ± 0.35 ^a^	22.45 ± 2.55 ^a^	48.97 ± 3.17

**Table 12:** Fertilization treatments applied in the experimental apple orchard, including treatment codes, row lines, application rates, and timing.

Treatment	Line	Description	Application Timing
T100	L7	Control—100% conventional fertilization	According to crop cycle
Myc2	L8	Mycoshell^®^ (2 tablets per tree)	At planting
Myc4	L9	Mycoshell^®^ (4 tablets per tree)	At planting
iNM6	L10	Kiplant iNmass^®^ (6 L ha^−1^ annually)	March annually
iNM12	L11	Kiplant iNmass^®^ (12 L ha^−1^ annually)	March annually
Allg6	L12	Kiplant All-Grip^®^ (6 L ha^−1^ annually)	March annually
Allg12	L13	Kiplant All-Grip^®^ (12 L ha^−1^ annually)	March annually
T70	L14	Control—70% conventional fertilization	According to crop cycle

**Table 13:** Annual fertilization plan applied in the experimental apple orchard (2020–2022).

Year	Treatment	15-7-25 (kg/ha)	Asflow MO (L/ha)	Ammonium Nitrate (kg/ha)	N (kg/ha)	P (kg/ha)	K (kg/ha)
2020	T100	300	200	17.85	50	21	75
	T70/Bio	210	140	12.5	35	14.7	52.5
2021	T100	300	200	17.85	45	21	75
	T70/Bio	210	140	12.5	31.5	14.7	52.5
2022	T100	360	200	58	70.2	25.2	90
	T70/Bio	252	140	40.6	49.2	17.6	63

T70/Bio includes the T70 control and all biofertilizer treatments (Myc2, Myc4, iNM6, iNM12, Allg6, and Allg12).

## Data Availability

The data presented in this study are available upon request from the corresponding author.

## Abbreviations

The following abbreviations are used in this manuscript:

Allg6	Kiplant All-Grip® biofertilizer, 6 L ha−1
Allg12	Kiplant All-Grip® biofertilizer, 12 L ha−1
ANOVA	Analysis of Variance
B	Boron
BBI	Bearing index
Ca	Calcium
CEC	Cation exchange capacity
C/N	Carbon-to-nitrogen ratio
CV	Canopy Volume
Cu	Copper
DAFB	Days after full bloom
DM	Dry matter
EC	Electrical conductivity
ETo	Reference evapotranspiration
Fe	Iron
INIAV	National Institute for Agrarian and Veterinary Research, I.P., Portugal
iNM6	Kiplant iNmass® biofertilizer, 6 L ha−1
iNM12	Kiplant iNmass® biofertilizer, 12 L ha−1
K	Potassium
KMO	Kaiser–Meyer–Olkin Test
Mg	Magnesium
Myc2	Mycoshell® biofertilizer, 2 tablets per tree
Myc4	Mycoshell® biofertilizer, 4 tablets per tree
N	Nitrogen
OM	Organic matter
P	Phosphorus
PCA	Principal Component Analysis
PGPR	Plant growth-promoting rhizobacteria
pH (H2O)	Soil pH measured in water
pH (CaCl2)	Soil pH measured in 0.01 M calcium chloride solution
RC	Rotated Component (from PCA)
RH	Relative humidity
Rs	Solar radiation
S	Sulfur
T100	100% conventional mineral fertilization
T70	70% conventional mineral fertilization
TCSA	Trunk cross-sectional area
TH	Tree height
TSS	Total soluble solids
UF	Fertilization units
Zn	Zinc

## References

[1] Savci S. (2012). An agricultural pollutant: Chemical fertilizer. Int. J. Environ. Sci. Dev..

[2] Aktar W., Sengupta D., Chowdhury A. (2009). Impact of pesticides use in agriculture: Their benefits and hazards. Interdiscip. Toxicol..

[3] Chittora D., Parveen T., Yadav J., Meena B.R., Tripta J., Jain T., Sharma K. (2023). Harmful impact of synthetic fertilizers on growing agriculture and environment. Glob. J. Pharmaceu. Sci..

[4] Bhattacharyya P.N., Jha D.K. (2012). Plant growth-promoting rhizobacteria (PGPR): Emergence in agriculture. World J. Microbiol. Biotechnol..

[5] Iqbal A., Fahad S., Danish S., Datta R., Saud S., Lichtfouse E., Fahad S., Danish S., Datta R., Saud S., Lichtfouse E. (2023). Biofertilizers to improve soil health and crop yields. Sustainable Agriculture Reviews 61.

[6] Zhang X., Zhang L., Liu J., Shen Z., Liu Z., Gu H., Hu X., Yu Z., Li Y., Jin J. (2025). Biofertilizers enhance soil fertility and crop yields through microbial community modulation. Agronomy.

[7] Mishra P., Dash P.R. (2014). Rejuvenation of biofertilizer for sustainable agriculture economic development. Cons. J. Sustain. Dev..

[8] Alori E.T., Glick B.R., Babalola O.O. (2017). Microbial phosphorus solubilization and its potential for use in sustainable agriculture. Front. Microbiol..

[9] Zhao G., Zhu X., Zheng G., Meng G., Dong Z., Baek J.H., Jeon C.O., Yao Y., Xuan Y.H., Zhang J. (2024). Development of biofertilizers for sustainable agriculture over four decades (1980–2022). Geogr. Sustain..

[10] Brahmaprakash G.P., Sahu P. (2012). Biofertilizers for sustainability. J. Indian Inst. Sci..

[11] Saini P. (2015). Mechanisms of plant growth promotion by rhizobacteria. J. Pure Appl. Microbiol..

[12] Figiel S., Rusek P., Ryszko U., Brodowska M.S. (2025). Microbially enhanced biofertilizers: Technologies, mechanisms of action, and agricultural applications. Agronomy.

[13] Malusá E., Vassilev N. (2014). A contribution to set a legal framework for biofertilisers. Appl. Microbiol. Biotechnol..

[14] Harman G.E., Uphoff N. (2019). Symbiotic root-endophytic soil microbes improve crop productivity and provide environmental benefits. Scientifica.

[15] Alayafi A.A.M., Alharbi B.M., Abdulmajeed A.M., Alnusaire T.S., Alrashidi A.A., Al-Balawi S.M., Khalaf A.H., Alghanem S.M.S., Al Zoubi O.M., Soliman M.H. (2025). Arbuscular mycorrhizal fungi symbiosis enhances growth, nutrient uptake, and oil quality in sunflower–pumpkin under intercropping systems. Front. Plant Sci..

[16] Singh B.K., Hu H.-W., Macdonald C.A., Xiong C. (2025). Microbiome-facilitated plant nutrient acquisition. Cell Host Microbe.

[17] Lisek J., Sas-Paszt L., Mika A., Lisek A. (2022). The response of weeds and apple trees to beneficial soil microorganisms and mineral fertilizers applied in orchards. Agronomy.

[18] Wang L., Yang F., E Y., Yuan J., Raza W., Huang Q., Shen Q. (2016). Long-term application of bioorganic fertilizers improved soil biochemical properties and microbial communities of an apple orchard soil. Front. Microbiol..

[19] Mosa W., Paszt L., Frąc M., Trzciński P. (2015). The role of biofertilization in improving apple productivity—A review. Adv. Microbiol..

[20] Mosa W.F.A.E.-G., Sas-Paszt L., Frąc M., Trzciński P., Treder W., Klamkowski K. (2018). The role of biofertilizers in improving vegetative growth, yield and fruit quality of apple. Horticulturae.

[21] Leão de Sousa M., Gonçalves M. (2022). Application of biofertilizers in ‘Gala’ apple orchards at planting: Consequences in mineral content, agronomic and physiological performance. Acta Hortic..

[22] Du T., Hu Q., He H., Mao W., Yang Z., Chen H., Sun L., Zhai M. (2023). Long-term organic fertilizer and biofertilizer application strengthens the associations between soil quality index, network complexity, and walnut yield. Eur. J. Soil Biol..

[23] Claro A.M., Fonseca A., Fraga H., Santos J.A. (2024). Future agricultural water availability in mediterranean countries under climate change: A systematic review. Water.

[24] Ferreira S.S., Seifollahi-Aghmiuni S., Destouni G., Ghajarnia N., Kalantari Z. (2022). Soil degradation in the european mediterranean region: Processes, status and consequences. Sci. Total Environ..

[25] Kumar D., Purakayastha T., Shivay Y. (2015). Long-Term Effect of Organic Manures and Biofertilizers on Physical and Chemical Properties of Soil and Productivity of Rice-Wheat System. Int. J. Bio-Resour. Stress Manag..

[26] Comissão Europeia Farm to Fork Strategy—Action Plan. https://food.ec.europa.eu/document/download/472acca8-7f7b-4171-98b0-ed76720d68d3_en?filename=f2f_action-plan_2020_strategy-info_en.pdf.

[27] Elshaer I.A., Azazz A.M.S., Hassan S.S., Fayyad S. (2023). Farm-to-Fork and sustainable agriculture practices: Perceived economic benefit as a moderator and environmental sustainability as a mediator. Sustainability.

[28] Matías J., Rodríguez M.J., Carrillo-Vico A., Casals J., Fondevilla S., Haros C.M., Pedroche J., Aparicio N., Fernández-García N., Aguiló-Aguayo I. (2024). From ‘Farm to Fork’: Exploring the potential of nutrient-rich and stress-resilient emergent crops for sustainable and healthy food in the mediterranean region in the face of climate change challenges. Plants.

[29] Nawaz R., Parkpian P., Garivait H., Anurakpongsatorn P., Delaune R., Jugsujinda A. (2012). Impacts of Acid Rain on Base Cations, Aluminum, and Acidity Development in Highly Weathered Soils of Thailand. Commun. Soil Sci. Plant Anal..

[30] Nguyen B., Vo D.L., Tong X., Nguyen T., Ngo Q., Van B., Go V. (2020). Ho Chi Minh City District. The Interactive Effects of Natural Factor and Pollution Source on Surface Water Quality in the Lower Mekong River Basin, Southwestern Vietnam. Water Resour..

[31] Mota M., Martins M.J., Policarpo G., Sprey L., Pastaneira M., Almeida P., Maurício A., Rosa C., Faria J., Martins M.B. (2022). Nutrient Content with Different Fertilizer Management and Influence on Yield and Fruit Quality in Apple cv. Gala. Horticulturae.

[32] USDA-NRCS Salinity Management in Soils for Irrigated Crops. United States Department of Agriculture—Natural Resources Conservation Service, 2012. https://www.nrcs.usda.gov/.

[33] WSU Orchard Soil Management and Compost Considerations. Washington State University Extension, 2023. https://treefruit.wsu.edu/.

[34] Yang M., Zhou D., Hang H., Chen S., Liu H., Su J., Lv H., Jia H., Zhao G. (2024). Effects of Balancing Exchangeable Cations Ca, Mg, and K on the Growth of Tomato Seedlings (*Solanum lycopersicum* L.) Based on Increased Soil Cation Exchange Capacity. Agronomy.

[35] Wu S.C., Cao Z.H., Li Z.G., Cheung K., Wong M. (2005). Effects of biofertilizer containing N-fixer, P and K solubilizers and AM fungi on maize growth: A greenhouse trial. Geoderma.

[36] Pang F., Li Q., Solanki M.K., Wang Z., Xing Y.X., Dong D.F. (2024). Soil phosphorus transformation and plant uptake driven by phosphate-solubilizing microorganisms. Front. Microbiol..

[37] Zhang F., Wang N., Zhao C., Yang L., Zhao X., Gao H., Zhang F., Wang H., Huang N. (2025). Enhancing black soil fertility and microbial community structure via microbial agents to reduce chemical fertilizer dependency: A strategy to boost maize yield. Agronomy.

[38] Marzouk S.H., Kwaslema D.R., Omar M.M., Mohamed S.H. (2025). Harnessing the power of soil microbes: Their dual impact in integrated nutrient management and mediating climate stress for sustainable rice crop production: A systematic review. Heliyon.

[39] Wu W., Ma B. (2015). Integrated nutrient management (INM) for sustaining crop productivity and reducing environmental impact: A review. Sci. Total Environ..

[40] Fan Y., Hao X., Ding R., Kang S. (2020). Soil water and nitrogen dynamics from interaction of irrigation and fertilization management practices in a greenhouse vegetable rotation. Soil Sci. Soc. Am. J..

[41] Warren C. (2009). Why does temperature affect relative uptake rates of nitrate, ammonium and glycine: A test with *Eucalyptus pauciflora*. Soil Biol. Biochem..

[42] Devi V., Sidhu A., Gosangi A., Sethi M. (2023). Effect of Low-Temperature Stress on Plant Performance and Adaptation to Temperature Change.

[43] White P.J., Brown P.H. (2010). Plant nutrition for sustainable development and global health. Ann. Bot..

[44] Møller I., Taiz L., Zeiger E., Murphy A. (2018). Plant Physiology and Development.

[45] Li S.-X., Wang Z.-H., Malhi S.S., Li S.-Q., Gao Y.-J., Tian X.-H. (2009). Nutrient and Water Management Effects on Crop Production, and Nutrient and Water Use Efficiency in Dryland Areas of China. Advances in Agronomy.

[46] Ntsomboh Ntsefong G., Gabriel M.S.T., Namuene K., Dzeufouo M., Tabi K. (2025). Biofertilizers: An Integrated Approach to Improving Soil Fertility, Plant Nutrition, Forest and Environmental Sustainability. OALib.

[47] Kumar S., Diksha, Sindhu S.S., Kumar R. (2022). Biofertilizers: An Ecofriendly Technology for Nutrient Recycling and Environmental Sustainability. Curr. Res. Microb. Sci..

[48] Dzvene A.R., Chiduza C. (2024). Application of Biofertilizers for Enhancing Beneficial Microbiomes in Push–Pull Cropping Systems: A Review. Bacteria.

[49] Ferreira E.T., Caetano L.E.S., Candido J.M.B., Cechin I., da Silva G.H.R. (2025). Enhancing Plant Growth and Photosynthesis with Biofertilizers from Sewage Treatment. Agronomy.

[50] Saied El Sayed A.B., Bakry O.A., Nofal M.A., Abo Horish M. (2024). Effectiveness of Biofertilizers Foliar Application on Yield and Quality Traits of Flax (*Linum usitatissimum* L.). Oil Crop Sci..

[51] Zhang B., Zhang H., Lu D., Cheng L., Li J. (2023). Effects of Biofertilizers on the Growth, Leaf Physiological Indices and Chlorophyll Fluorescence Response of Spinach Seedlings. PLoS ONE.

[52] Koularmanis K., Tsouvaltzis P., Siomos A. (2025). Impact of Elevated Temperature and Solar Radiation on Broccoli (*Brassica oleraceae* var. *italica* Plenck) Cultivation. Horticulturae.

[53] Dong S.X., Davies S.J., Ashton P.S., Bunyavejchewin S., Supardi M.N., Kassim A.R., Tan S., Moorcroft P.R. (2012). Variability in Solar Radiation and Temperature Explains Observed Patterns and Trends in Tree Growth Rates across Four Tropical Forests. Proc. Biol. Sci..

[54] Ali A., Jabeen N., Chachar Z., Chachar S., Ahmed S., Ahmed N., Laghari A.A., Sahito Z.A., Farruhbek R., Yang Z. (2025). The Role of Biochar in Enhancing Soil Health & Interactions with Rhizosphere Properties and Enzyme Activities in Organic Fertilizer Substitution. Front. Plant Sci..

[55] Kumar P., Chandel R.S., Verma S.C., Sharma N., Saini S., Bishist R., Lata S. (2025). Biostimulation through Natural Biological Inputs on Fruiting, Nutrient Availability and Rhizosphere Microbiome in Legume Intercropped ‘Sweet Charlie’ Strawberry (*Fragaria × Ananassa Duch.*). BMC Plant Biol..

[56] Kavadia A., Omirou M., Fasoula D., Ioannides I.M. (2020). The Importance of Microbial Inoculants in a Climate-Changing Agriculture in Eastern Mediterranean Region. Atmosphere.

[57] Díaz-Rodríguez A.M., Parra Cota F.I., Cira Chávez L.A., García Ortega L.F., Estrada Alvarado M.I., Santoyo G., de los Santos-Villalobos S. (2025). Microbial Inoculants in Sustainable Agriculture: Advancements, Challenges, and Future Directions. Plants.

[58] Hedhly A., Hormaza J., Herrero M. (2003). The Effect of Temperature on Stigmatic Receptivity in Sweet Cherry (*Prunus avium* L.). Plant Cell Environ..

[59] Strømme C., Sivadasan U., Nissinen K., Lavola A., Randriamanana T., Julkunen-Tiitto R., Nybakken L. (2019). Interannual Variation in UV-B and Temperature Effects on Bud Phenology and Growth in *Populus tremula*. Plant Physiol. Biochem..

[60] Abdel-Sattar M., Almutairi K.F., Aboukarima A.M., El-Mahrouky M. (2021). Impact of Organic Manure on Fruit Set, Fruit Retention, Yield, and Nutritional Status in Pomegranate (*Punica granatum* L. “Wonderful”) under Water and Mineral Fertilization Deficits. PeerJ.

[61] Chen C., Nelson A.S., Shaw T., Kimsey M. (2022). Effects of Fertilization on the Growth Dominance of Inland Northwest Forests of the United States. Forest Ecosyst..

[62] Baïram E., le Morvan C., Delaire M., Buck-Sorlin G. (2019). Fruit and Leaf Response to Different Source–Sink Ratios in Apple, at the Scale of the Fruit-Bearing Branch. Front. Plant Sci..

[63] Bergh O. (1990). Effect of Time of Hand-Thinning on Apple Fruit Size. S. Afr. J. Plant Soil.

[64] Fischer G., Almanza-Merchán P., Ramírez F. (2012). Source–Sink Relationships in Fruit Species: A Review. Rev. Colomb. Cienc. Hortic..

[65] Lévesque M., Walthert L., Weber P. (2016). Soil Nutrients Influence Growth Response of Temperate Tree Species to Drought. J. Ecol..

[66] Wolf M.K., Wiesmeier M., Macholdt J. (2023). Importance of Soil Fertility for Climate-Resilient Cropping Systems: The Farmer’s Perspective. Soil Secur..

[67] Morariu P.A., Mureșan A.E., Sestras A.F., Dan C., Andrecan A.F., Borsai O., Militaru M., Mureșan V., Sestras R.E. (2025). The Impact of Cultivar and Production Conditions on Apple Quality. Notulae Bot. Horti Agrobot. Cluj-Napoca.

[68] Figueiredo A., Silva A., Tavares C., Pastaneira M., Melo J., Rodrigues C., Machado A., Antunes M., Cruz C., Marques da Silva J. (2025). Influence of Orchards Fertilization Management and Post-Harvest Storage Time on *Malus domestica* cv. ‘Gala’ Fruit Volatiles and Quality Parameters. J. Food Compos. Anal..

[69] McGlone V.A., Kawano S. (1998). Firmness, Dry-Matter and Soluble-Solids Assessment of Postharvest Kiwifruit by NIR Spectroscopy. Postharvest Biol. Technol..

[70] Gómez-Devia L., Nevo O. (2024). Effects of Temperature Gradient on Functional Fruit Traits: An Elevation-for-Temperature Approach. BMC Ecol. Evol..

[71] Roussos P.A. (2024). Climate Change Challenges in Temperate and Sub-Tropical Fruit Tree Cultivation. Encyclopedia.

[72] Bacelar E., Pinto T., Anjos R., Morais M.C., Oliveira I., Vilela A., Cosme F. (2024). Impacts of Climate Change and Mitigation Strategies for Some Abiotic and Biotic Constraints Influencing Fruit Growth and Quality. Plants.

[73] Keller M. (2010). Managing Grapevines to Optimise Fruit Development in a Challenging Environment: A Climate Change Primer for Viticulturists. Aust. J. Grape Wine Res..

[74] Fioravanço J.C., Czermainski A.B.C. (2018). Biennial bearing in apple cultivars. Rev. Ceres..

[75] Krasniqi A.L., Blanke M.M., Kunz A., Damerow L., Lakso A.N., Meland M. (2017). Alternate bearing in fruit tree crops: Past, present and future. Acta Hortic..

[76] Kottek M., Grieser J., Beck C., Rudolf B., Rubel F. (2006). World Map of the Köppen–Geiger Climate Classification Updated. Meteorol. Z..

[77] Instituto Português do Mar e da Atmosfera (IPMA) Climate Normal—Alcobaça/Fruticulture Station Vieira Natividade, 1991–2020. https://www.ipma.pt/bin/file.data/climate-normal/cn_91-20_ALCOBACA_E_FRUTICULTURA.pdf.

[78] Lambers H., Barrow N.J. (2020). P_2_O_5_, K_2_O, CaO, MgO, and basic cations: Pervasive use of references to molecules that do not exist in soil. Plant Soil.

[79] (2006). Soil Quality—Pretreatment of Samples for Physico-Chemical Analysis, 2nd ed.

[80] Milosevic T., Milosevic N., Mladenovic J. (2019). Tree vigor, yield, fruit quality, and antioxidant capacity of apple (*Malus × domestica* Borkh.) influenced by different fertilization regimes: Preliminary results. Turk. J. Agric. For..

[81] Direção-Geral de Agricultura e Desenvolvimento Rural (DGADR) (2011). Normas Técnicas para a Produção Integrada de Pomóideas.

[82] Maroco J. (2014). Análise Estatística Com O SPSS.

